# Sex differences in romantic love: an evolutionary perspective

**DOI:** 10.1186/s13293-025-00698-4

**Published:** 2025-02-24

**Authors:** Adam Bode, Severi Luoto, Phillip S. Kavanagh

**Affiliations:** 1 Australian Capital Territory, The Australian National University, Canberra, ACT Australia; 2 Independent Researcher, Auckland, New Zealand; 3 Discipline of Psychology, Faculty of Health, University of Canberra, Bruce, ACT Australia; 4 Justice and Society, University of South Australia, Magill, SA Australia

**Keywords:** Romantic love, Romantic Love Survey 2022, Sex differences, Sexual dimorphism, Sexual selection, Young adults

## Abstract

**Background:**

Evolutionary selection pressures, most notably sexual selection, have created (and continue to sustain) many psychobehavioral differences between females and males. One such domain where psychobehavioral sex differences may be prominent is romantic love. The ways in which females and males may experience and express romantic love differently has been studied in psychology as well as in the arts down the ages; however, no studies have focused specifically on romantic love (i.e., passionate love) using validated measures of romantic love solely in people who are currently experiencing this form of love.

**Methods:**

This study investigated sex differences in features and aspects of romantic love among 808 young adults experiencing romantic love. Univariate and multivariate analyses were conducted to measure sex differences in the number of times participants had fallen in love, when they fell in love relative to when they started their romantic relationship (love progression), intensity of romantic love, obsessive thinking about a loved one, and commitment. Additional univariate comparisons were made for sex differences in Passionate Love Scale scores.

**Results:**

Univariate analyses showed that males had fallen in love a greater number of times than females. Males had also fallen in love more quickly than females. Females had higher intensity of romantic love, higher commitment, and higher obsessive thinking about a loved one than males. These findings remained robust in multivariate analyses, controlling for several variables believed to influence romantic love, with the exception of commitment, which was no longer significant when other variables were controlled for.

**Conclusions:**

The findings are considered with reference to the evolutionary theory of sexual selection. We suggest that the specific adaptive challenges faced by females and males in the evolutionary history of romantic love may contribute to sex differences in romantic love. The findings shed light on contemporary sex differences in romantic love, as well as the possible evolutionary history and evolutionary functions of romantic love.

**Supplementary Information:**

The online version contains supplementary material available at 10.1186/s13293-025-00698-4.

## Plain Language Summary

Some studies have investigated differences in romantic love (i.e., passionate love) between females and males, but none have done so using validated measures of romantic love while focusing solely on people who are currently in love. Our participants were all young adults (aged 18-25) from 33 different countries mainly in Europe, North America, and South Africa. All participants were currently in love. We compared females and males on (1) the number of times they had ever been in love, (2) when they fell in love compared to when they started their romantic relationship, (3) the intensity of romantic love, (4) how much they obsessed about their partner, and (5) their level of commitment to their partner. Some small differences were found between females and males for all these variables, but when we took into account other things that may influence romantic love, we found that sex differences generally diminished. We discuss the findings with reference to one evolutionary theory related to romantic love.

**Supplementary Information:**

The online version contains supplementary material available at 10.1186/s13293-025-00698-4.

## Highlights

- Males fall in love slightly more often than females do.
- Males fall in love about one month earlier than females do.
- Females experience romantic love slightly more intensely than males do.
- Females in love experience obsessive thinking about their loved one more than males do.
- Females in love are slightly more committed than males are.
- Sex differences reduce at a multivariate level.
- Romantic love may have solved some different adaptive challenges for females and males.

**Supplementary Information:**

The online version contains supplementary material available at 10.1186/s13293-025-00698-4.

## Introduction

Men always want to be a woman’s first love. That is their clumsy vanity. We women have a more subtle instinct about these things. What (women) like is to be a man’s last romance. - Oscar Wilde

Romantic love is a common theme in literature [10], movies [80], and music [32]. It is both a great motivator for pair bonding and sexual union, as well as the most important preference for people when considering long-term romantic partners [28]. Lay conceptions of romantic love abound, including perceived differences between the sexes; however, minimal research has been undertaken specifically investigating sex differences in what most researchers call romantic love (i.e., passionate love). Some scientific attempts have been made to explain any differences in romantic love using evolutionary theory (see [4, 21, 44, 53, 102]). Romantic love is the basis for romantic relationship and family formation throughout most of the world (see [24]). Understanding romantic love is fundamental to understanding modern romantic relationships and human mating as well as the fundamental human motivation to form pair-bonds (see [12, 17]).

### Definition of romantic love

The scientific literature is imprecise and confusing in the terminology surrounding romantic love. Most biological psychologists and human behavioral scientists who study the topic refer to romantic love as a specific motivational state that occurs commonly at the early stages of romantic relationship [16, 17]. It can sometimes occur in the absence of a romantic relationship [22] or, less commonly, it can still be present after many years or decades of partnership [1]. It is characterized by particularly strong cognitions, emotions, and behaviors [41, 48] including obsessive thinking about a loved one [12] and sexual activity [18]. Some psychologists conflate the term “romantic love” with any type of love in romantic relationships, while other psychologists refer to it specifically as “early-stage romantic love” or “passionate love.” In this article, romantic love refers specifically to early-stage romantic love (i.e., passionate love). Lay people commonly refer to romantic love as “being in love.” Romantic love contrasts with companionate love, which is associated with pair bond maintenance [12], less intensity and passion, and feelings of companionship [50].

Bode and Kushnick [17] drew on a comprehensive review of the biology and psychology of romantic love to define romantic love as:

a motivational state typically associated with a desire for long-term mating with a particular individual. It occurs across the lifespan and is associated with distinctive cognitive, emotional, behavioral, social, genetic, neural, and endocrine activity in both sexes. Throughout much of the life course, it serves mate choice, courtship, sex, and pair-bonding functions. It is a suite of adaptations and by-products that arose sometime during the recent evolutionary history of humans (p. 21).

#### The role of romantic love in romantic relationships and human mating

Human mating strategies vary between short-term mating and long-term mating [26]. A short-term mating strategy involves one or a relatively small number of instances of sexual intercourse and is associated with one-night stands or short-term trysts. A long-term mating strategy involves prolonged engagement and commitment between partners and is associated with committed relationships, pair bonds, social monogamy, and marriage. Both strategies can result in offspring and both strategies have been, and continue to be, adaptive in certain circumstances and environments.

Romantic love is a motivational state, present in both sexes, that serves a number of reproductive functions, including mate choice, courtship, sex, and pair bonding [17]. It is associated with a range of neural and endocrine systems that each play distinct roles in various psychobehavioral facets of romantic love (e.g., bonding attraction, attachment, obsessive thinking, courtship attraction, and sexual desire; see [12]). Romantic love can manifest in childhood, but all its features are not present until after puberty [17]. It probably emerged by co-opting mother-infant bonding between 6 and 8 million years ago and 300,000 years ago [12].

Romantic love serves a mate choice function [17, 41]. Romantic love is often a precondition for long-term romantic relationship formation and continuation. As a rule, when an individual has fallen in love with someone, they are motivated to form a long-term relationship with that person (with the exception in some cases of infidelity). Romantic love is one of several mate choice mechanisms: Bode and Kushnick ([17]) noted that “[m]ate preferences are important because they may serve as a means of screening potential mates, while sexual desire and attraction operationalize these preferences, and romantic love crystalizes them” (p. 5).

Romantic love serves a courtship function [17, 41]. Some individuals fall in love before a romantic relationship has formed; in such cases, romantic love plays a role in motivating that individual to engage in courtship behaviors and to pursue their loved one. Even after a romantic relationship has formed, continued courtship, both proactive and receptive, takes place until a romantic relationship is fully formed.

Romantic love serves a sex function (at a proximal level) and a reproductive function (at an ultimate, evolutionary level; [17, 73]). Wanting to express one’s love to a loved one is one of the main reasons why both females and males have sex (fifth most common reason for females; eighth most common reason for males; [73, 74]). Individuals in love have sex with greater frequency than individuals in a romantic relationship not experiencing romantic love, and this increase in sexual frequency appears to be associated with the state of romantic love rather than its intensity [18]. The possible downstream evolutionary benefits of increased sexual intimacy include pregnancy [17].

Romantic love serves a pair bonding function [17, 42]. In particular, romantic love plays a role in pair bond formation [12]. Numerous biological processes involving dopamine, oxytocin, and opioids influence the creation, and transition to maintenance, of a pair bond (see [12]). Romantic love bonds partners together by creating shared understandings, emotions, and habits [50].

Pair bonding is ubiquitous among humans [85]. It is relatively rare among mammals (see [78]). Pair bonds tend to last more than one reproductive cycle [9] and in humans can vary in length from a period of months or years to a lifetime. Romantic love is one primary means through which pair bonds are formed [17]. The other means include arranged marriages or other forms of long-term commitment that may lack romantic love.

Understanding the evolution of romantic love has helped to shed light on the mechanisms underlying it and the functions it serves [12, 17]. For example, recent theory about the evolution of romantic love suggests that three systems found in mother-infant bonding (i.e., bonding attraction, attachment, and obsessive thinking) are also active in romantic love [12]. Combined with two additional reproductive systems (i.e., courtship attraction and sexual desire), these five systems work interdependently to serve a range of different functions (see [12]). These systems involve dopamine, oxytocin, serotonin, testosterone, and nerve growth factor circuitry [17].

Sex differences in romantic love have implications for the evolutionary functions of romantic love. For example, sex differences in romantic love may affect the degree and manner in which members of each sex select mates, engage in courtship, initiate or engage in sex, and form pair bonds or exit them. Such differences may be evident in the way and degree to which the biological mechanisms of romantic love are up-regulated or down-regulated. For example, blood levels of serotonin have been shown to be up-regulated in females but down-regulated in males [59], and this may be because there are sex-specific outputs regulated by the serotonin system in romantic love. There may be sex differences in the psychological underpinnings or behavioral expression of romantic love. However, the current state of scientific knowledge is limited when it comes to understanding sex differences in romantic love.

### What is known about sex differences in romantic love?

There have been studies investigating sex differences in romantic love, but none of them (to our knowledge) have used a sample of people exclusively experiencing romantic love. Most psychological studies have tended to use samples of individuals in a romantic relationship, some of whom are likely to be experiencing romantic love. Some, however, (e.g., [30]) also include participants not in a romantic relationship. This research has shed some light on sex differences in the intensity of romantic love, the timing of when males versus females proclaim “I love you,” the prevalence of unrequited love, and the number of times individuals have ever fallen in love. Two studies using samples of individuals experiencing romantic love have identified some neural and endocrine sex differences in romantic love [41, 59]. Gender is often used as a proxy for biological sex in both psychological and biological studies, and if gender is conceptualized as a female/male dichotomy in studies on romantic love, it can be seen to map on to biological sex with a high degree of fidelity (given the low prevalence of transgender individuals).

One study [30], which validated an Italian version of the Passionate Love Scale (PLS; [48]) in a sample of 784 Italians (57% of whom self-identified as being in love), compared males and females on total PLS scores. Females scored marginally higher on the PLS than males. This is consistent with the findings of an earlier study of 1,090 Texan undergraduate students [53]. That study asked “how much in love” participants were. The findings indicated that females scored higher than males did; however, the effect size was small (*R*^2^ = 0.02). The findings of these studies contradict other studies that found no sex differences in the intensity of romantic love (e.g., [83, 35, 48]) using samples that may have included participants experiencing romantic love.

There is good evidence that males express their love sooner than females do [4, 21, 47, 102]. This finding, based on people’s personal recall of who said “I love you” first in a current or former romantic relationship, has been demonstrated in university and community samples. Most studies have used US samples, but the general findings are internationally robust: a recent study [102] demonstrated that males are significantly more likely to say “I love you” first in samples from five of seven investigated countries (i.e., Brazil, Chile, Colombia, Poland, United Kingdom; cf. Australia, France). While the sample sizes were small for five of the seven countries (i.e., *n* < 100), the effect sizes ranged from moderate to large for all countries investigated (0.51 > *r* > .72).

To our knowledge, two studies have investigated sex differences for when females and males fall in love. The results are mixed. Galperin and Haselton [44] tested the prediction that “men fall in love more easily than do women” among 357 heterosexual American participants recruited from an online advertising site. That study asked participants when they fell in love relative to their partner (i.e., before, at the same time, after). No significant sex differences were found in the proportion of females or males that fell in love before their partner (male = 27%, female = 32%). Harrison and Shortall [47] asked 149 American university students who had previously been in a committed romantic relationship “[…] how long did it take you to realize you were in love?” in their most recent romantic relationship. Males reported falling in love more quickly than females did, and the effect size was moderate in magnitude (*d* = 0.48). The average duration for males was at the high end of “a few weeks” while for females it was at the low end of “a few months.”

We are aware of two studies that have investigated the prevalence of unrequited romantic love (romantic love where the loved one does not reciprocate). Galperin and Haselton [44] found that males had been in a romantic relationship with 69% of their “love objects,” whereas females had been in a romantic relationship with 80%. Hill et al. [54] asked 140 American university students to recall the number of times they had experienced unrequited love at three age groups (i.e., younger than 10, 10–15, 16–20) and found significant sex differences for the number of episodes of unrequited love (i.e., male > female) in the 16–20 age range.

There is evidence from two studies that males fall in love more often than females do, while one study did not find such a difference. Galperin and Haselton [44] found no sex differences in number of episodes of romantic love in their sample (*N* = 357), whereas Hendrick and Hendrick [53] found a very small (*R*^2^ approaching zero) but significant difference between females (2.50 episodes) and males (2.63 episodes) in their university sample. A third study [34] of 503 Spanish university students found that males had fallen in love a substantially greater number of times (3.06 episodes) on average than females had (2.16 episodes).

In an fMRI study of romantic love, Fisher et al. [41] reported that males showed greater activation than females in the right posterior dorsal insula, which is associated with penile turgidity and the viewing of beautiful faces, and in regions associated with the integration of visual stimuli. Another study by Langeslag et al. [59] found that females experiencing romantic love exhibited higher serum and plasma serotonin levels than males experiencing romantic love. In addition, females experiencing romantic love displayed higher serotonin plasma levels than female controls, whereas the opposite effect was found for males: males experiencing romantic love displayed lower serotonin levels than control males. This study was of high quality but would require replication as endocrinological studies are notoriously noisy and inconsistent (see [46]). Some other studies (e.g., [53, 56, 72, 75, 76]; see also [93]) have investigated sex differences in relation to the Love Attitude Scale’s [52] Eros subscale, which is sometimes conflated with romantic love (see [16]). We choose not to consider this literature as the Eros subscale was not developed to measure romantic love, and others have advised against using it to measure romantic love [16], despite it frequently being done [51].

### The role of evolution in creating and maintaining psychological sex differences

We follow a theory-driven top-down approach from evolutionary psychology [61] in forming the predictions of this study. This approach consists of, firstly, identifying a specific problem related to survival or reproduction that would have been consistently present in ancestral humans or in their environments. Secondly, it is necessary to articulate specific psychobehavioral predisposition(s) that would have (hypothetically) solved the adaptive problem. These include perceptual systems that detect cues related to the problem (inputs); computational (e.g., cognitive, hormonal) mechanisms (algorithms) that process these cues; and emotions, cognitions, and behaviors produced by these algorithms to solve the adaptive problem (outputs; [61]). Here, we focus our analysis on the psychobehavioral outputs that would have hypothetically been caused by sex-specific adaptive problems. These adaptive problems are related to reproduction.

Behavioral differences between females and males are present not only in humans, but Darwinian sex roles have been observed across the animal kingdom [55]. In line with the Darwin–Bateman paradigm, anisogamy imposes stronger sexual selection on males, resulting in female-biased parental care and male-biased sexual dimorphism and lower parental investment [55, 62]. As a result of anisogamy, each sex faces different adaptive problems. Males face the adaptive problem of gaining sexual access to fertile females. Females face the adaptive problem of securing a male that is willing and capable of providing resources for the offspring (this adaptive problem is somewhat mitigated in some contemporary societies that have implemented policies that make it viable for females to be single parents; nevertheless, this altered fitness landscape has not been present long enough, evolutionarily speaking, to erase females’ preference for high-status males who would be viable providers for their offspring). The downstream effects of anisogamy and differential sexual selection pressures on psychobehavioral sex differences in humans – including mate choice and mating strategies – have been thoroughly studied in evolutionary psychology (e.g., [6, 27, 63, 65]), but less so in the context of romantic love.

Evolution by natural selection is the only known natural process that creates higher degrees of functional order in living organisms or counteracts the unavoidable increase in disorder that would otherwise ensue [94, 95]. Any functional organization in undomesticated organisms that is greater than expected by chance ultimately results from natural selection [25, 61, 94, 95]. In this respect, humans are no different, and still subject to the same evolutionary processes as other species [55, 64]. Although there are many factors that may influence sex differences in human behavior – such as genetics, prenatal sex hormone exposure, local ecology, individual development, social history, and phylogeny – evidence from various sources indicates that evolutionary forces, including sexual selection, sexual differentiation of the mammalian brain, sexual division of labor, and their interactions, ultimately drive psychobehavioral sex differences in humans (see [64]; for a review). Although some researchers have argued that socialization into gender roles causes sex differences in humans, this hypothesis becomes less plausible when biological, developmental, neuroscientific, and cross-national evidence is considered more broadly (reviewed in [64]). Furthermore, evolutionary processes pre-date social conceptualizations of gender roles by several million years. Therefore, a full explanation of how social influences might affect sex differences in cognition and behavior would need to account for how evolutionary processes act as precursors to gender roles, whilst also partialing out any biological influences on sex differences in cognition and behavior [7, 55, 64, 70].

Psychological sex differences evolved in circumstances where females and males faced different adaptive challenges [6, 23]. There are few areas in which the challenges faced by females and males differ more greatly than in mating and reproduction. Males have faced the challenges of securing access to females whilst also being exposed to potential paternity uncertainty through female extra-pair copulations. Females have faced the problem of securing a reliable or replenishable supply of resources to sustain them (and their offspring) through pregnancy and lactation by identifying and selecting males that are both able and willing to invest in a long-term partnership [23, 62]. Both sexes face the added task of assessing a partner’s genetic quality, health during development, and current condition (including physical, mental, and behavioral traits) [89]—though sex differences can also be expected in the relative importance of these traits in mate choice.

As romantic love serves reproductive functions (i.e., mate choice, courtship, sex, and pair bonding), and as male and female fitness landscapes are vastly different owing to anisogamy [63, 96], evolutionary theory would predict that substantial differences may exist between the sexes in the frequency, intensity, and psychobehavioral features or consequences of romantic love [23]. This would be the case in domains where females and males faced different adaptive challenges, such as courtship, sexual behavior, commitment, and maximizing sex-specific fitness landscapes that arise from anisogamy and internal gestation. Consequently, we would assume that when aspects of romantic love solved different adaptive challenges for females and males, larger sex differences will be present. In circumstances where females and males faced the same or similar adaptive challenges, psychological sex differences will be very small or non-existent. The discussion section of the current study considers the results in the context of sexual selection 36].

The tendency for males to confess their love before females is considered the most common sex difference in romantic love in terms of evolutionary theory. Drawing on theory about mating strategies from sociobiology, Brantley et al. ([21]) first considered this tendency in relation to male efforts to coax females into having sex with them. This idea has been developed since then, and researchers have made a compelling case that one function of romantic love is that it acts as a kind of commitment device (see [4, 24, 43]; see also [102]). In essence, the idea is that because females bear greater costs of pregnancy and child-birth than males do [96], females seek males who will commit to them, ensuring the provision of resources including food and protection throughout the period of pregnancy and early lactation (see [17, 62]). It has been suggested that romantic love is an “honest signal” of deep emotional involvement [86]. As a result, males may say “I love you” before females because they fall in love sooner (e.g., [47]) or because they are attempting to deceive a female into believing they have committed to them [21]. The former seems likely to be more common; after all, much of the evidence of males saying “I love you” before females comes from people who had been in a (potentially committed) romantic relationship, so romantic love is likely to have been a genuine feature underlying the males’ verbal expressions of love.

### The current study

#### Predictions

The current study is the first to specifically investigate sex differences in romantic love using a relatively large cross-cultural sample and validated measures. As a result, it provides the best evidence to date of whether sex differences exist in some aspects of romantic love. Given that romantic love serves reproductive purposes, and psychological sex differences are expected in domains related to reproduction [23], combined with extant evidence of sex differences in aspects of romantic love, we predicted that:

1. Males will have fallen in love more often than females (because of anisogamy and sex differences in parental investment, males should have a lower threshold to become reproductively invested in a potential mate);
2. Males will have fallen in love sooner than females (because the male fitness landscape favors quantity of potential mates over quality, whereas the opposite is true for females);
3. Females will express a greater intensity of romantic love than males (because females have more to gain from a committed long-term relationship than males do).

We had no predictions about the associations between biological sex and two other measures of romantic love (obsessive thinking about a loved one and commitment). We also conducted additional exploratory analyses to further investigate sex differences in romantic love using The Passionate Love Scale (Hatfield & Sprecher, 48).

#### Controls in multivariate analyses

We selected a broad range of controls for use in our multivariate analyses based on theory and literature. Individual-level controls included participants’ age, months they had been in love, and days since they had sex. Population-level control variables included sex ratio (number of males per 100 females) and gender inequality. Sex ratios are known to impact reproductive and social behaviors [67]. Gender inequality could influence sex differences in the expression of romantic love [103]. The number of times ever in love could feasibly represent a trade-off between frequency and intensity or may otherwise influence characteristics of romantic love. Months in love could influence the intensity and form of romantic love, as different trajectories of features of love (i.e., passion, intimacy, and commitment) have been suggested to wax or wane over the period when an individual is in a romantic relationship [45, 90]. Days since sex could also have some impact upon the experience of romantic love as sexual activity in the previous 48 h has been shown to influence feelings about one’s romantic relationship [71].

We used measures of some features of romantic love as controls in some analyses. This was done because we understand the features of romantic love (e.g., number of times in love, love progression, intensity of romantic love, obsessive thinking about a loved one, and commitment) to be causally related, and previous research has found some strong associations between some of these features. Specifically, previous research [15] has found that obsessive thinking and commitment are significantly associated with the intensity of romantic love in a sample very similar to the present study drawn from the Romantic Love Survey 2022 [14]. We believe these are bi-directionally causally related as the intensity of romantic love may represent a global construct that comprises the individual features of romantic love (i.e., obsessive thinking and commitment), and the intensity of romantic love may indicate the degree of activation or expression of multiple features that are acting in concert (see [12, 17]).

We also believe that the modern sex differences in number of times an individual has ever fallen in love and when they fall in love relative to when a romantic relationship commenced may be indicative of evolved sex differences. This could also be associated with specific biological mechanisms (such as genetics, sex hormones, and life history strategy) that influence other features of romantic love (i.e., intensity, obsessive thinking, and commitment). In essence, we suspected that a common third factor (e.g., the biological, psychological, or social mechanisms that cause romantic love) may influence both of these factors (i.e., number of times fallen in love and timing of falling in love) and other features of romantic love. We recognize that including romantic love-related variables in regression models as controls may conflate the findings to some degree and we therefore provide the results of the relevant analyses without these romantic love-related variables in the body text, while the models with those control variables are presented in the Supplementary Material.

#### Preregistration

This study was preregistered [13] after a small amount of the data had been collected and viewed. The same analysis conducted in this study was undertaken on a sample of 199 participants from the Romantic Love Survey 2022 [14] prior to preregistration in October 2022 to facilitate an abstract to present at a conference. The predictions and methods remained the same. However, two insightful peer reviewers of the current article highlighted errors with our hypotheses and predictions, as well as with our planned approach to test these hypotheses and predictions.

In the preregistration we failed to indicate the direction of any anticipated association between sex and features of romantic love (an obvious oversight) and made predictions that were not testable using the methods we proposed. Specifically, we predicted the magnitude of effect sizes for the anticipated associations between biological sex and features of romantic love, but more sophisticated statistical methods than proposed would be required to test these predictions. We acknowledge these errors and have revised our predictions and analyses accordingly. We now indicate the direction of the association (which are obvious from the literature review provided above) in the form of predictions and have removed references to the anticipated magnitude of effect sizes of associations. Contrary to the preregistration, we also decided to omit the use of digit ratios as a control variable. This is because a deeper reflection of the relevant research on digit ratios, prenatal sex hormone exposure, and sexual differentiation of the brain (e.g., [7, 64, 65, 70]) suggested that digit ratio (as a proxy of prenatal sex hormone exposure) is not a “nuisance variable” to be controlled for in sex difference analyses but rather a potential mediator between biological sex and psychobehavioral outcome measures. Deviations from preregistration are appropriate when mistakes have been made [58]; however, the preregistration still adds value to this study by demonstrating that the selection of main variables and methods used in our analyses was made prior to undertaking the analyses.

## Methods

### Participants

Participants were 808 English-speaking young adults (female = 47.77%) from the publicly available Romantic Love Survey 2022 [14] who self-identified as being in love. The majority of the female participants (94.6%, *n* = 365) identified as women, while 5.4% (*n* = 21 females) identified as neither men or women (most of them identified as non-binary). Nevertheless, these participants were analyzed as biological females. Most male participants (98.1%, *n* = 414) identified as men, while 0.7% (*n* = 3 males) identified as women, and 1.2% (*n* = 5 males) identified as neither men nor women. All of these participants were analyzed as biological males in this study. The Romantic Love Survey 2022 was collected online using Prolific [81] between October and December 2022. Participants were aged 18–25 and resided in 33 different countries, primarily in North America, Europe, and South Africa. The survey was administered in English. There was a high proportion of students in the sample (69.93%) but this was expected given the age of participants and the platform used. Supplementary Table 1 presents the characteristics of the entire sample used in this study. We follow Bode and Kowal [16] for sample characteristics reporting to maximize comparability with other studies and the assessment of generalizability. Supplementary Table 2 shows the participants’ country of residence.

All participants answered “yes” to the question: “Are you currently in love with someone you have a romantic relationship with (dating, committed relationship, or married/de facto)?” Only participants who had been in love for 23 months or less were included in the analyses. This is because two years is a likely period of time in which individuals experience early-stage romantic love rather than long-term romantic love, a state which differs psychologically, mechanistically, and functionally from early-stage romantic love [1, 2, 79]. All participants had been in a romantic relationship for less than 48 months. Participants were also only included if they scored above 130 on the Passionate Love Scale (PLS; [48]), which the originators of the scale have indicated is associated with having, at least, “tepid, infrequent passion” [49]. This approach to maximizing the likelihood that participants are experiencing romantic love rather than companionate love has been used previously [15]. Data for one intersex participant was not included in the analysis to facilitate the use of sex as a binary variable (see [38]; for a discussion on the binary nature of sex). Three cases were missing a total of four data points and all data for these cases were removed.

### Measures

#### Biological sex

Biological sex was measured using a simple question asking, “What is your biological sex?” Data were coded as 1 (female) or 2 (male).

#### Number of times in love

Number of times in love was collected using a simple question asking, “How many times have you ever been in love (including this time)?”

#### Love progression

Participants answered questions about (i) the length of time they had been in love and (ii) the duration of their romantic relationship (indicated by when they at least started dating). Participants were asked “How long have you been in love with the person you love?” and “How long have you had a romantic relationship with the person you love (since you started dating or seeing the person romantically)?” A variable, *Love progression*, assessing the sequence of romantic love and romantic relationship commencement (difference in months), was computed by subtracting the time in love from relationship duration. Scores below 0 indicate that an individual fell in love before their romantic relationship commenced, scores of 0 indicate that an individual fell in love at the same time as their romantic relationship commenced, and positive scores indicate that an individual fell in love after their romantic relationship had commenced.

#### Measures of romantic love

The psychological characteristics of romantic love were measured using the PLS-30 [48], percent of time thinking about loved one, and the five commitment items from the Triangular Love Scale Short-form (TLS-15; [57]). The *Intensity of romantic love* was measured with the PLS-30, which is a 30-item measure of the cognitive, emotional, and behavioral characteristics of romantic love. It assesses the intensity of romantic love. Each item recorded scores by assessing agreement with statements on a nine-point Likert scale (1 = *not at all true*; 9 = *definitely true*). PLS-30 has been used cross-culturally [40] and is the most commonly used measure of romantic love in studies investigating the biological mechanisms of romantic love [16]. Cronbach’s alpha for the PLS-30 in this sample was 0.936. Obsessive thinking about a loved one is indicative of romantic love [12, 20, 59]. Percent of waking hours thinking about a loved one (*Obsessive thinking*) was measured on a sliding scale ranging from 0 to 100%. Commitment is one component of love (Sternberg [90]) and romantic love serves as a “commitment device” (see [17, 24, 43]). The TLS-15 *commitment* subscale is a five-item measure of commitment truncated from the full 45-item TLS commitment subscale [57]. The original TLS-15 version used a five-point scale for each item, but the Romantic Love Survey 2022 retained the traditional TLS nine-point scale. Each item records scores by assessing agreement with statements on a nine-point Likert scale (1 = *not at all*; 9 = *extremely*). Cronbach’s alpha for the TLS-15 commitment subscale items in this sample was 0.899.

#### Control variables

Five control variables were used in regression analyses: (i) *age*, (ii) *sex ratio*, (iii) *gender inequality*, (iv) *months in love*, and (v) *days since sex*. Measures of romantic love (number of times in love, love progression, intensity of romantic love, obsessive thinking, and commitment) were also used as controls in additional analyses (see Supplementary Materials). Age was assessed by asking participants to indicate their age in years. Nation-level sex ratio (number of males per 100 females at birth) was taken for ages 15–49 in 2021 (i.e., we used sex ratios at birth for this age cohort, meaning that the data included sex ratio at birth between the years 1972–2006) from the United Nations [97]. Gender Inequality Index scores for 2021 [98] were sourced for the country in which participants resided. Participants indicated how many months they had been in love. Participants were asked “How long since the last time you had sex with the person you love? “Sex” is however you choose to interpret it.” Responses ranged from “today” to “more than 7 days ago.”

#### Procedure

Means, standard deviations (for continuous variables), and percentages (for the categorical variables) were calculated for variables used in the regression analyses, and *t*-tests were conducted for all romantic love variables with biological sex as the independent variable. Pearson’s correlations among all variables were calculated. To test prediction one, a hierarchical linear regression was conducted with sex predicting number of times in love, while controlling for relevant variables. To test prediction two, a hierarchical linear regression was conducted with sex predicting love progression, while controlling for relevant variables. To test prediction three, a hierarchical linear regression was conducted with biological sex predicting intensity of romantic love. Two additional exploratory analyses were undertaken by conducting two hierarchical linear regressions with sex predicting obsessive thinking and commitment, while controlling for relevant variables. We identified possible outliers by calculating Mahalanobis distances [66] using a cutoff of *p* < .001 [82].

In the final exploratory analysis, we undertook 30 *t*-tests comparing mean scores for females and males on each of the 30 items of the PLS-30. Effect sizes and confidence intervals were generated using Cohen’s *d*. In presenting the results, we emphasize effect sizes and their confidence intervals instead of *p*-values [5, 11], owing to the exploratory nature of these item-specific PLS-30 analyses. *P*-values have unknown diagnostic value in exploratory research, and using them in this context can incorrectly suggest that hypotheses are being tested instead of generated [5, 77]. We followed Hyde [84], see also [38], in interpreting the magnitude of Cohen’s *d* effect sizes so that sex difference effects were considered small when 0.11 < *d* ≤ 0.35; moderate when 0.36 < *d* ≤ 0.65; large when 0.66 < *d* ≤ 0.99; and very large when *d* ≥ 1.00.

## Results

Means, standard deviations, and percentages for variables used in the regression analyses, as well as *t*-tests for all romantic love variables, are shown in Table 1. All romantic love variable means differed significantly by sex. Table 2 presents the zero order correlations for all variables used in the regression analyses. All assumptions for all regressions were met.

**Table 1:** Means, standard deviations, *t*-statistics, and univariate sex effect sizes of variables used in this study

	Female				Male								95% CI	
Variable	*M*	*SD*	*n*	%	*M*	*SD*	*n*	%	*t*	*df*	*p*	Cohen’s *d*	Lower	Upper
Sex			386	100.00			422	100.00						
Number of times in love	2.32	1.37			2.64	1.54			−3.15	806	0.002	−0.22	−0.36	−0.08
Love progression	1.92	4.44			0.98	4.29			3.07	806	0.002	0.22	0.08	0.36
Intensity of romantic love	213.41	29.90			206.37	32.05			3.22	806	0.001	0.23	0.09	0.37
Obsessive thinking	53.89	21.94			44.58	22.22			5.99	806	< 0.001	0.42	0.28	0.56
Commitment	37.11	7.09			36.09	6.56			2.11	806	0.035	0.15	0.01	0.29
Age	22.28	1.86			22.06	1.86								
Sex ratio	101.74	2.88			102.03	3.27								
Gender inequality	0.15	0.12			0.15	0.12								
Months in love	8.49	6.26			7.77	5.67								
Days since sex	3.63	2.86			3.36	2.90								

Positive *d*-values indicate higher values in females; negative *d*-values indicate higher values in males

*n* = 808

**Table 2:** Zero order correlations among variables used in the regression analyses

Variable		1	2	3	4	5	6	7	8	9	10	11
1	Sex (male)	1	0.11^**^	−0.11^***^	−0.11^***^	−0.21^***^	−0.07^*^	−0.06	0.05	0.00	−0.06	0.00
2	Number of times in love		1	−0.17^***^	−0.11^***^	−0.08^*^	−0.13^***^	0.05	−0.03	0.15^***^	−0.09^**^	0.05
3	Love progression			1	−0.11^***^	−0.04	−0.10^**^	0.07	−0.01	0.00	−0.09^**^	−0.02
4	Intensity of romantic love				1	0.47^***^	0.61^***^	−0.04	−0.02	0.11^***^	0.14^***^	−0.03
5	Obsessive thinking					1	0.34^***^	0.01	−0.07^*^	0.18^***^	0.06	−0.06
6	Commitment						1	−0.02	−0.03	0.08^*^	0.24^***^	−0.12^***^
7	Age							1	−0.01	−0.02	0.07^*^	−0.06^*^
8	Sex ratio								1	−0.23^***^	−0.02	0.01
9	Gender inequality									1	0.02	0.01
10	Months in love										1	−0.02
11	Days since sex											1

^*^p < .05; ^**^p < .01; ^***^p < .001

### Biological sex and number of times in love

According to prediction 1, males would have fallen in love more often than females. This was tested using a hierarchical linear regression in which sex predicted number of times in love after controlling for age, sex ratio, gender inequality, and months in love.

The hierarchical linear regression predicting number of times in love revealed that at Step 1, control variables significantly contributed to the regression model, *F*(4, 803) = 7.321, *p* < .001 and accounted for 3.04% of the variation in number of times in love. Adding sex to the regression model (Step 2) explained an additional 1.00% of the variation in number of times in love and this change in adjusted *R*^2^ was significant, *F*(1, 802) = 9.793, *p* = .002. Males had fallen in love a greater number of times than females. Table 3 presents the regression statistics for this analysis.

**Table 3:** Hierarchical regression model of Number of Times in Love

									95% CI	
	*R*^*2*^	Adjusted *R*^*2*^	Δ Adjusted *R*^*2*^	*b*	*SE*	β	*t*	*p*	Lower	Upper
Step 1	0.035^***^	0.030^***^								
Age				0.049	0.027	0.062	1.77	0.076	−0.01	0.10
Sex ratio				0.003	0.017	0.007	0.19	0.849	−0.03	0.04
Gender inequality				1.902	0.440	0.154	4.32	< 0.001	1.04	2.77
Months in love				−0.025	0.009	−0.100	−2.87	0.004	−0.04	−0.01
Step 2	0.047^***^	0.041^***^	0.010^**^							
Age				0.053	0.027	0.067	1.95	0.052	0.00	0.11
Sex ratio				0.001	0.017	0.002	0.05	0.964	−0.03	0.03
Gender inequality				1.887	0.437	0.153	4.32	< 0.001	1.03	2.75
Months in love				−0.023	0.009	−0.094	−2.71	0.007	−0.04	−0.04
Biological sex (male)				0.318	0.102	0.108	3.13	0.002	0.12	0.12

*n* = 808; ^**^p < .01; ^***^p < .001

### Biological sex and love progression

According to prediction 2, males would have fallen in love sooner than females. This was tested using a hierarchical regression in which sex predicted love progression controlling for age, sex ratio, and gender inequality. Love progression was a variable constructed by subtracting months in love from relationship duration (months) whereby negative scores indicated participants had fallen in love prior to starting their romantic relationships, scores of 0 indicated that they fell in love at the same time as they started their romantic relationship, and positive scores indicated they fell in love after starting their romantic relationship.

The hierarchical linear regression predicting love progression revealed that at Step 1, control variables did not contribute significantly to the regression model, *F*(3, 804) = 1.172, *p* = .319 and accounted for < 0.01% of the variation in love progression. Adding sex to the regression model (Step 2) explained an additional 0.96% of the variation in love progression and this change in adjusted *R*^2^ was significant, *F*(1, 803) = 8.753, *p* = .003. Males had fallen in love sooner than females. Table 4 presents the regression statistics for this analysis. Supplementary Table 3 present the results of the regression analysis with number of times in love added as a control variable.

**Table 4:** Hierarchical regression model of love progression

									95% CI	
	*R*^*2*^	Adjusted *R*^*2*^	Δ Adjusted *R*^*2*^	*b*	*SE*	β	*t*	*p*	Lower	Upper
Step 1	0.004	0.001								
Age				0.154	0.083	0.065	1.86	0.064	−0.01	0.32
Sex ratio				−0.011	0.051	−0.008	−0.22	0.823	−0.11	0.09
Gender inequality				0.068	1.334	0.002	0.05	0.959	−2.55	2.69
Step 2	0.015^*^	0.010^*^	0.010^**^							
Age				0.140	0.083	0.059	1.69	0.091	−0.02	0.30
Sex ratio				−0.004	0.051	−0.003	−0.08	0.934	−0.11	0.10
Gender inequality				0.106	1.328	0.003	0.08	0.937	−2.50	2.71
Biological sex (male)				−0.912	0.308	−0.104	−2.96	0.003	−1.52	−0.31

*n* = 808; ^*^p < .05; ^**^p < .01

We note the mean love progression score for females was 1.92 months after romantic relationship formation whereas for males it was 0.98 months, indicating that males fell in love approximately one month sooner than females did. The median love progression score was 1.00 (one month after romantic relationship formation) for females and 0.00 (the same time as romantic relationship formation) for males, suggesting that males fell in love sooner than females. The mode for both females and males was 0.00. We ran a post-hoc analysis comparing the proportion of participants who fell in love prior to the formation of a romantic relationship. Results indicated that a greater proportion of males (30.09%) than females (19.69%) fell in love prior to the onset of a romantic relationship, χ^2^(1, 808) = 11.604, *p* < .001.

### Biological sex and intensity of romantic love

According to prediction 3, females would express a greater intensity of romantic love than males. This was tested with a hierarchical linear regression that predicted intensity of romantic love using sex as the independent variable, while controlling for age, sex ratio, gender inequality, months in love, and time since sex.

The hierarchical linear regression predicting the intensity of romantic love revealed that at Step 1, control variables contributed significantly to the regression model, *F*(5, 802) = 5.771, *p* < .001 and accounted for 2.87% of the variation in intensity of romantic love. Adding sex to the regression model (Step 2) explained an additional 1.06% of the variation in intensity of romantic love and this change in adjusted *R*^2^ was significant, *F*(1, 801) = 9.840, *p* = .002. Females scored higher on the intensity of romantic love than males. Table 5 presents the regression statistics for this analysis. Supplementary Table 4 presents the results of the regression analysis with additional romantic love-related controls.

**Table 5:** Hierarchical regression model of intensity of romantic love

									95% CI	
	*R*^*2*^	Adjusted *R*^*2*^	Δ Adjusted *R*^*2*^	*b*	*SE*	β	*t*	*p*	Lower	Upper
Step 1	0.035^***^	0.029^***^								
Age				−0.913	0.585	−0.054	−1.56	0.119	−2.06	0.23
Sex ratio				0.114	0.360	0.011	0.32	0.752	−0.59	0.82
Gender inequality				27.481	9.364	0.105	2.93	0.003	9.10	45.86
Months in love				0.754	0.182	0.144	4.14	< 0.001	0.40	1.11
Days since sex				−0.326	0.377	−0.030	−0.86	0.388	−1.07	0.41
Step 2	0.046^***^	0.039^***^	0.011^**^							
Age				−1.010	0.582	−0.060	−1.74	0.083	−2.15	0.13
Sex ratio				0.166	0.359	0.016	0.46	0.643	−0.54	0.87
Gender inequality				27.791	9.313	0.106	2.98	0.003	9.51	46.07
Months in love				0.723	0.182	0.138	3.98	< 0.001	0.37	1.08
Days since sex				−0.331	0.375	−0.031	−0.88	0.377	−1.07	0.40
Biological sex (male)				6.790	2.165	−0.109	−3.14	0.002	−11.04	−2.54

*n* = 808; ^**^p < .01, ^***^p < .001

### Biological sex and obsessive thinking

We conducted a hierarchical linear regression that predicted obsessive thinking using sex as the independent variable, while controlling for age, sex ratio, gender inequality, months in love, and time since sex.

The hierarchical linear regression predicting obsessive thinking revealed that at Step 1, control variables accounted for 3.28% of the variation in obsessive thinking. Adding sex to the regression model (Step 2) explained an additional 4.01% of the variation in obsessive thinking. Females reported thinking about their loved one more than males. Table 6 presents the regression statistics for this analysis. Supplementary Table 5 presents the regression results with additional romantic love-related variables added as controls.

**Table 6:** Hierarchical regression model of obsessive thinking

							95% CI		
	*R*^*2*^	Adjusted *R*^*2*^	Δ Adjusted *R*^*2*^	*b*	*SE*	β	Lower	Upper	
Step 1	0.039	0.033							
Age				0.011	0.422	0.001	−0.82	0.84	
Sex ratio				−0.229	0.260	−0.031	−0.74	0.28	
Gender inequality				31.901	6.753	0.168	18.65	45.16	
Months in love				0.202	0.131	0.053	−0.06	0.46	
Days since sex				−0.484	0.272	−0.062	−1.02	0.05	
Step 2	0.080	0.073	0.040						
Age				−0.121	0.413	−0.100	−0.93	0.69	
Sex ratio				−0.158	0.255	−0.022	-0.66	0.34	
Gender inequality				32.320	6.612	0.170	19.34	45.30	
Months in love				0.159	0.129	0.042	−0.09	0.41	
Days since sex				−0.492	0.266	−0.063	−1.01	0.03	
Biological sex (male)				−9.174	1.537	−0.203	−12.19	−6.16	

*n* = 808

### Biological sex and commitment

We conducted a hierarchical linear regression that predicted commitment score using sex as the independent variable controlling for age, sex ratio, gender inequality, months in love, and time since sex. The hierarchical linear regression predicting commitment revealed that at Step 1, control variables accounted for 7.30% of the variation in number of times in love. Adding sex to the regression model (Step 2) explained an additional 0.28% of the variation in commitment. Females scored higher on commitment than males. Table 7 presents the regression statistics for this analysis.

**Table 7:** Hierarchical regression model of commitment

								95% CI		
	*R*^*2*^	Adjusted *R*^*2*^	Δ Adjusted *R*^*2*^	*b*	*SE*	β	*t*	Lower	Upper	
Step 1	0.079	0.073								
Age				−0.173	0.125	−0.047	−1.39	−0.42	0.07	
Sex ratio				−0.019	0.077	−0.008	−0.24	−0.17	0.13	
Gender inequality				4.396	2.001	0.077	2.20	0.47	8.32	
Months in love				0.274	0.039	0.239	7.03	0.20	0.35	
Days since sex				−0.273	0.081	−0.115	−3.39	−0.43	−0.11	
Step 2	0.083	0.076	0.003							
Age				−0.185	0.125	−0.051	−1.48	−0.43	0.06	
Sex ratio				−0.012	0.077	−0.005	−0.16	−0.16	0.14	
Gender inequality				4.435	1.998	0.077	2.22	0.51	8.36	
Months in love				0.270	0.039	0.236	6.93	0.19	0.35	
Days since sex				−0.274	0.080	−0.115	−3.40	−0.43	−0.12	
Biological sex (male)				−0.855	0.464	−0.063	−1.84	−1.77	0.06	

*n* = 808

### Exploratory analyses of biological sex and the PLS-30

The exploratory analysis compared scores on 30 individual items of the PLS-30 using independent sample *t*-tests. Effect sizes were calculated for each sex difference comparison using Cohen’s *d*. As shown in Table 8, small sex differences were identified for despair, jealous, no one else, yearn, want, affection, tender, lonely, signs, and touch, and moderate magnitude sex differences were identified for know me, and bubbly. Table 8 provides the means and standard deviations for females and males for each of the PLS-30 items as well as the test statistics, Cohen’s *d*, and 95% confidence intervals for the point estimate. Positive *d*-values indicate higher scores for females; negative *d*-values indicate higher scores for males.

**Table 8:** Means, standard deviations, *t*-statistics, degrees of freedom, and Cohen’s *d*s for sex differences for each PLS-30 item

		Female		Male					95% CI	
PLS-30 item		*M*	*SD*	*M*	*SD*	*t*	*df*	Cohen’s *d*	Lower	Upper
1	Rollercoaster	5.24	2.34	5.28	2.30	−0.27	806	−0.02	−0.16	0.12
2	Despair	6.97	2.05	6.56	1.97	2.91	806	0.21	0.07	0.34
3	Trembles	6.40	2.06	6.34	1.88	0.42	806	0.03	−0.11	0.17
4	Studying	6.89	1.97	6.61	1.94	2.05	806	0.14	0.01	0.28
5	Obsessive	5.42	2.27	5.51	2.18	−0.58	806	−0.04	−0.18	0.10
6	Happy	8.02	1.19	7.79	1.37	2.51	806	0.18	0.04	0.32
7	Rather	7.49	1.61	7.27	1.68	1.87	806	0.13	−0.01	0.027
8	Jealous	7.84	1.80	7.30	1.93	4.06	806	0.29	0.15	0.42
9	No one else	5.45	2.59	5.89	2.27	−2.59	806	−0.18	−0.32	−0.04
10	Yearn	7.44	1.50	7.02	1.70	3.75	806	0.26	0.13	0.40
11	Want	8.19	1.11	7.79	1.28	4.71	806	0.33	0.19	0.47
12	Forever	6.83	1.91	6.71	1.85	0.87	806	0.06	−0.08	0.20
13	Melt	7.37	1.60	7.14	1.70	1.98	806	0.14	0.00	0.28
14	Affection	7.70	1.38	7.25	1.60	4.38	806	0.31	0.17	0.44
15	Perfect	7.16	1.76	7.16	1.67	0.01	806	0.00	−0.14	0.14
16	Happiest	7.46	1.64	7.32	1.56	1.19	806	0.08	−0.05	0.22
17	Body	7.95	1.27	7.72	1.36	2.52	806	0.18	0.04	0.32
18	Tender	8.00	1.25	7.66	1.34	3.77	806	0.27	0.13	0.40
19	On mind	7.10	1.57	6.88	1.65	1.94	806	0.14	0.00	0.27
20	Lonely	7.10	1.88	6.74	1.89	2.71	806	0.19	0.05	0.33
21	Concentrate	5.27	2.33	5.09	2.20	1.16	806	0.08	−0.06	0.22
22	Know me	7.69	1.42	7.12	1.65	5.20	806	0.37	0.23	0.51
23	Complete	7.63	1.48	7.51	1.40	1.18	806	0.08	−0.05	0.22
24	Signs	7.41	1.54	6.93	1.70	4.15	806	0.29	0.15	0.43
25	Concerns	7.72	1.36	7.65	1.42	0.67	806	0.05	−0.09	0.19
26	Bubbly	7.52	1.27	7.08	1.46	5.89	806	0.41	0.28	0.55
27	Touch	7.91	1.27	7.52	1.47	3.98	806	0.28	0.14	0.42
28	Existence	6.01	2.28	5.79	2.22	1.34	806	0.09	−0.04	0.23
29	Attraction	7.58	1.37	1.48	1.43	1.06	806	0.07	−0.06	0.21
30	Depressed	6.54	2.05	6.25	2.08	2.01	806	0.14	0.00	0.28

Positive *d*-values indicate higher scores for females; negative *d*-values indicate higher scores for males

## Discussion

This study tested three predictions about sex differences in various aspects of romantic love: the number of times participants had fallen in love; when individuals fell in love relative to when they started their romantic relationships (love progression); and the intensity of romantic love. The predictions were drawn from sexual selection theory as well as from studies investigating sex differences in romantic love. We also conducted exploratory analyses to investigate sex differences in two additional features of romantic love (obsessive thinking and commitment) and any sex differences in items of a commonly used measure of the cognitive, emotional, and behavioral features of romantic love (i.e., PLS-30). Univariate sex differences on all five romantic love outcome variables (Table 1) were statistically significant (and in the predicted direction for the three variables for which predictions were provided). Using multivariate analyses, all three of our predictions were supported. Of the two additional analyses investigating the multivariate association between biological sex and features of romantic love, we found that females had higher obsessive thinking than males (the largest association of all outcome variables investigated) but no significant sex difference in commitment was found. Our exploratory analyses of PLS-30 items identified some small and moderate univariate sex differences in the features of romantic love.

The data provided support for prediction 1: the univariate analysis indicated that males had fallen in love a significantly greater number of times than females. This is consistent with previous research [53]. A very small significant positive association of male sex with number of times in love was identified in the multivariate analysis. Control variables explained a greater proportion of the variance in number of times in love than did biological sex.

The data provided support for prediction 2: the univariate analysis indicated that males had fallen in love significantly earlier than females. This is consistent with previous research [47]. A very small significant negative association of male sex with love progression was identified in the multivariate analysis. Biological sex explained more of the variance in love progression than did control variables. Post-hoc analysis indicated that a greater proportion of males than females fell in love before they commenced their romantic relationship.

The data provided support for prediction 3: the univariate analysis indicated that females experienced romantic love more intensely than males. This is consistent with previous research [30, 53]. There was a very small significant negative association of male sex with intensity of romantic love in the multivariate analysis. Control variables explained more of the variance in the intensity of romantic love than did biological sex.

The univariate analysis indicated that females experienced obsessive thinking about their loved one more than males did. This was the largest univariate sex differences of any outcome variable considered. This result remained robust in the multivariate analysis, which showed that males had notably lower obsessive thinking scores than females did. This was the largest multivariate association among the outcome variables considered. Biological sex explained more variance in obsessive thinking than control variables did.

The univariate analysis indicated that females experienced higher commitment than males. This was the smallest univariate sex difference among the outcome variables considered. This difference did not exist at a multivariate level, however, with no significant association between biological sex and commitment being found.

In our exploratory analysis of the PLS-30 items, small and moderate univariate sex differences were found for mostly emotional features of romantic love.

In conclusion, it is reasonable to ask whether the sex differences identified in this study are meaningful, whether sexual selection played a role in their emergence, and if any alternative theories might explain these sex differences.

### Where do meaningful sex differences occur in romantic love?

Univariate and multivariate sex differences were identified for the number of times ever in love, love progression, intensity of romantic love, and obsessive thinking. The univariate sex differences were all small (except for obsessive thinking, which had a moderate effect size; see Table 1) and these tended to reduce in magnitude at a multivariate level. That these sex differences were only moderate or small does not contradict the hypothesis that they are a result of evolutionary processes underlying distinct adaptive problems faced by males and females. Take, for example, sex differences in mate preferences (e.g., [23, 100]). At a univariate level, most of the commonly cited sex differences (i.e., preferences for health, kindness, intelligence, physical attractiveness, and financial prospects in a potential mate) are relatively small in magnitude [100]. However, when considered in concert with each other, these differences represent a meaningful difference between the sexes, with strong sex-predictive effect [33]. The same may be true of small and very small sex differences in various aspects of romantic love.

Of note, the largest sex difference in a feature of romantic love existed for obsessive thinking. Females thought about their loved one about 54% of their waking hours whereas males thought about their loved one 44% of waking hours. This difference is substantial, and a lay person would likely notice such a difference in themselves (vs. their partner) if experiencing romantic love. This raises questions about the functions of obsessive thinking and what adaptive challenges it helps to overcome (discussed below).

Small univariate and very small multivariate sex differences were identified for love progression (when an individual fell in love relative to when they started their relationship). The difference was about one month, with females falling in love on average about two months after starting a romantic relationship and males falling in love on average about one month after starting a romantic relationship. It was also demonstrated that a larger proportion of males than females had fallen in love before a romantic relationship had commenced. Falling in love one month earlier is practically meaningful. Falling in love one month earlier provides males with a greater opportunity to use romantic love to promote courtship (see [41]), to demonstrate romantic love as an honest signal of commitment [86], and to say “I love you” first [102]. All of this is consistent with the well documented sex difference in partner selection, with females being choosier than males [96], even in the context of mutual mate choice [92]. A small univariate or very small multivariate sex difference is sufficient to result in a plethora of downstream responses to adaptive demands by one or the other sex. According to this line of reasoning, a female would be less likely to fall in love before a male has shown adequate behavioral signs of commitment, which are the signal that typically enable the female to fully fall in love.

### Did sexual selection play a role in the evolution of sex differences in romantic love?

Prior research has established that psychobehavioral sex differences are common in domains related to reproduction [23]. This is because reproduction is a domain in which females and males commonly faced (and still face) different adaptive challenges. The role of sexual selection is understood to be predominant in the development of such psychobehavioral sex differences [6, 62, 64]. Romantic love serves several functions related to reproduction [17] and therefore it was anticipated that meaningful sex differences in various psychobehavioral facets of romantic love would be found, given that each sex faced different adaptive challenges in their evolutionary history.

An increased frequency of falling in love and earlier timing in which males fall in love may have been a means of overcoming the male-specific adaptive problem of needing to court and demonstrate commitment. In the evolutionary history of humans, females may have been more likely to choose suitors who fell in love first, who were motivated to court, and who provided an honest signal of commitment.

The sex difference in the intensity of romantic love is less clear-cut. The sex difference in the intensity of romantic love equates to only a 1% difference on PLS scores. It appears rather unlikely that sex differences in intensity of romantic love would be under direct sexual selection (i.e., that mate choice would favor different levels of romantic love in a sex-specific manner), or that mate choice would fully explain sex differences in the intensity of romantic love. Rather, it is more likely that sex differences in the intensity of romantic love arise because of other factors, such as anisogamy and differential parental investment, which might favor somewhat different levels of intensity of romantic love in males and females.

Something similar can be said for obsessive thinking about a loved one. If obsessive thinking did not affect behavior, it would also be unlikely that one sex or the other would select mates based on the percentage of time their potential mate thought about them. So, what behavioral outcomes result from obsessive thinking? The literature is scant on this topic. Despite being highlighted in both major theoretical contributions to understanding the evolution of romantic love [12, 41], surprisingly little research has been undertaken on the phenomenon of obsessive thinking (see [8, 20, 68, 69, 39 for exceptions), and no one has provided a likely explanation of its functions in romantic love (although note the suggestion of [68]). Notably, however, obsessive thinking is positively associated with commitment, intensity of romantic love (Table 2), and frequency of sexual activity (with the latter correlation being stronger in females than in males; [19]), which implies that it is one of the cognitive mechanisms underlying important behavioral aspects of romantic love.

Romantic love solved one fundamental challenge faced by both females and males in our evolutionary history – survival of the mother, fetus, and offspring in the early stage of life (see [12]). While small differences exist in various features of romantic love, and some of these may be the result of sexual selection acting on different adaptive problems faced by females and males, it is undeniable that the primary reproductive function of romantic love when it emerged and throughout the subsequent evolution was successful reproduction and the survival of viable offspring into adulthood. According to this line of thinking, romantic love may initially serve the function of a commitment device whereby a male shows they are committed to a female, providing the female with a signal that allows her to become emotionally, physically, and reproductively invested in the male. This hypothesis is supported by the finding that males fall in love – and express their love – sooner than females (Table 1), as well as by a positive correlation (stronger in females than in males) between frequency of sex and three facets of romantic love: intensity, commitment, and obsessive thinking [19]. Thus, romantic love would lead (or would have evolutionarily led) from the identification of a suitable partner to commitment, copulation, and reproduction.

### A comment on the causal relationship between different features of romantic love

As indicated in the Introduction, we included several variables related to romantic love in the regression models as controls because we believed they were bi-directionally correlated with each other. There is support for this notion in most models (see Supplementary Materials). The intensity of romantic love was significantly associated with both obsessive thinking and commitment. Obsessive thinking was associated with commitment in a bivariate analysis (Table 2) but not when other variables were controlled for in a multivariate analysis (Supplementary Tables 5 and 6). This indicates that the relationship between obsessive thinking and commitment is either mediated or confounded by other variables. This should be considered in future research investigating the features of romantic love.

There was little evidence supporting our notion that the number of times an individual had ever fallen in love and when an individual fell in love relative to when they started their relationship might be associated with other features of romantic love because of a common third factor influencing both (i.e., biological, psychological, or social mechanisms of romantic love). Love progression (i.e., when individuals fell in love relative to when they started their romantic relationship) was significantly associated with the intensity of romantic love, suggesting a possible causal relationship via the individual differences in the biological, psychological, and social mechanisms that cause romantic love. Number of times ever in love was not associated with any of the outcome variables (although it was approaching significance for commitment). This may suggest that some of the mechanisms that influence some aspects of romantic love may also influence other aspects, but there may be other mechanisms that influence specific aspects, and not others. This may seem obvious, but to our knowledge, this study provides the first empirical evidence of this hypothesis.

### Insights into the interaction between evolution and the social environment

In our study, gender inequality was positively associated with number of times ever in love (Table 3), intensity of romantic love (Table 5), obsessive thinking (Table 6), and commitment (Table 7), even when controlling for other variables. This indicates that in countries with higher gender equality, people experience fewer instances of romantic love, and less obsessive thinking about and commitment to the person with whom they are in love, as well as less intense romantic love, than in countries with lower gender equality. Though these cross-national analyses are only preliminary (for example, we did not address statistical problems arising from spatial autocorrelation; see [31]; nor did we have large samples from all the countries included in the analyses), they suggest cross-national variation in certain facets of romantic love. This suggests that there is cross-national variance in either biological (e.g., genetic), ecological (e.g., harshness, life history variation), or cultural underpinnings [3, 37, 62, 63, 99] that result in cross-national variation in various aspects of romantic love. However, we emphasize that this remains to be determined with larger samples with greater global coverage and appropriate methodology [31].

### Future research

This study provides impetus for future research. A representative study of number of times in love would prove useful because it could take into account the impact of age on number of times in love. To analyze love progression in greater detail, it would be useful to also collect data on how long an individual has known their loved one and whether they experienced a crush or an acute courtship attraction episode prior to the onset of romantic love. Combining such data with additional data about sexual activity initiation and when individuals said “I love you” would prove incredibly informative. There are other means of assessing the intensity of romantic love (e.g., the Triangular Love Scale; [57, 91]; Infatuation and Attachment Scale; [60]) than PLS-30, and future research would benefit from using these measures alongside the PLS-30 to see if differences in results arise. There may also be opportunities to assess the contribution of biological sex to individual components of romantic love suggested by the theory of co-opting mother infant bonding (e.g., Infatuation [59]; Attachment [60]; obsessive thinking; love progression; and Sexual Desire [88]). Analyzing the biological, ecological, or cultural underpinnings that may contribute to sex differences in romantic love would be another avenue for future research. For instance, a recent study found that cross-national differences in modernization and collectivism are associated with cross-national differences in some aspects of romantic love, such as intimacy [87]. Such findings warrant further investigation, particularly considering the current findings implying that cross-national differences in gender inequality may covary with cross-national differences in romantic love. The current results showed that gender equality is associated with *less* commitment, intensity of romantic love, and obsessive thinking as well as *fewer* instances of romantic love.

### Limitations

While this study has been able to provide insights into the possible sex-specific evolutionary functions of romantic love, it is not without limitations. First, all participants were recruited online, speak fluent English, and reside in OECD countries plus South Africa. Additionally, they were generally highly educated, and most were current students. All were aged between 18 and 25 and were therefore not representative of the variety of humans. As a result, the generalizability of this study is somewhat limited (see [16]). The ability to draw evolutionary inferences from the current data is also limited, given the fact that the data were cross-sectional and the data did not include populations with ancestral lifestyles. Including populations with ancestral or near-ancestral lifestyles would have been beneficial for being able to rule out the hypothesis that the sex differences observed in this study may have somehow arisen from the significant cultural and/or environmental (e.g., lifestyle) changes that have taken place since the advent of agriculture.

A particularly noteworthy consideration in this respect is the emergence of contemporary lifestyles that are characteristic of large populations living in towns and cities rather than the small-scale populations that were the status quo during much of human evolution. These shortcomings are nevertheless somewhat mitigated inasmuch as the current findings are consistent and consilient with other evolutionarily salient findings regarding sex differences in contemporary humans (see e.g., [6, 55, 64] for more detailed discussions). Furthermore, the sex ratio data were provided at the level of nations, lacking the kind of granularity that would be required for more accurate estimates of how this variable may influence human mating. After all, sex ratios may substantially vary within countries, e.g., between different cities or between rural and urban areas, and nation-level data will not be accurate at the level of individuals residing in different parts of a country (see e.g., [101]; who reported that the correlation between city-level overall sex ratio and country-level sex ratio at birth was very low, *r* = .16). As the sex ratio data included sex ratios at birth for the years 1972–2006, it did not account for possible sex ratio changes that might have occurred because of emigration.

We also note that 27.10% of participants in our study identified with a non-heterosexual sexual orientation (see Supplementary Table 1). Given recent evidence that romantic love shows some psychobehavioral differences in different sexual orientation groups for both females and males [19], sex differences may be more pronounced when only heterosexual males and females are compared with each other; after all, non-heterosexual individuals create additional variation in the data, which somewhat muddles the sex differences between heterosexual males and females. It may therefore be important to ask not only whether there are sex differences in romantic love, but whether those differences are more substantial when only heterosexual males and females are compared with one another.

Our exploratory analysis comparing all variables on the PLS-30 by sex was conducted without a priori predictions, and the risk of type I error was substantially increased. The results of those analyses should therefore be considered only as preliminary findings that can be used as a guide for future confirmatory research. In relation to the analysis of love progression, we recognize that we are relying on participants’ recall, which may be imprecise. Nonetheless, this study is the first to specifically investigate the role of biological sex in a broad range of romantic love characteristics focusing solely on people experiencing romantic love. The findings of the exploratory analyses have generated ideas about the evolution of romantic love and demonstrate that a bottom-up approach to the science of romantic love can be useful when knowledge is sparse.

We do note, however, that there is a possibility that some of the participants in this study were not experiencing romantic love and were in fact experiencing companionate love. This is suggested by the fact that some participants scored relatively low on the PLS and had been in a romantic relationship for more than 2 years. We took steps to try to minimize the likelihood of including people experiencing companionate love (i.e., by including only those self-reporting being “in love” for 23 months or less and those scoring above 130 on the PLS). Nonetheless, there is evidence that people can confuse companionate love for romantic love [29] and readers should take this possibility into account as it may have added some additional variance to the models.

## Conclusion

This study investigated sex differences in romantic love. Small sex differences were found for number of times in love, love progression, commitment, and intensity of romantic love in univariate analyses, while the sex difference in obsessive thinking was of moderate magnitude, with females having higher scores on this trait. The findings are important because they are the first to study the topic in such detail. The findings support specific theories about the evolution of romantic love and the evolution of sex differences in romantic love. The results provide more support for perspectives on the evolutionary processes influenced by, and causing, romantic love.

### Perspectives and significance

This is the first study to investigate sex differences in a sample of people exclusively experiencing romantic love. At the univariate level, differences were identified between females and males in relation to number of times ever experiencing romantic love, when they fell in love relative to when they started their romantic relationship, the intensity of romantic love, obsessive thinking about a loved one, commitment, and particular features of romantic love (i.e., specific items of PLS-30). A key finding is that males fall in love on average one month earlier than females, and this may account for why males tend to say “I love you” first. These sex difference tended to decrease in multivariate analyses. This study has generated theory about the evolutionary history and evolutionary functions of romantic love. This is an important study applying evolutionary theory to a vital and understudied aspect of human mating. One of the strengths of the study is that participants were drawn from 33 different countries.

## Supplementary Information

Supplementary material 1

## Data Availability

The Romantic Love Survey 2022 data and materials are available at 10.15139/S3/WBVMFG. Code is available from the authors upon request. This study was preregistered: https://osf.io/acz34.

## References

[1] Acevedo BP, Aron A. Does a long-term relationship kill romantic love? Rev Gen Psychol. 2009;13(1):59–65. 10.1037/a0014226.

[2] Acevedo BP, Aron A, Fisher HE, Brown LL. Neural correlates of long-term intense romantic love. Soc Cognit Affect Neurosci. 2012;7(2):145–59. 10.1093/scan/nsq092. 21208991 10.1093/scan/nsq092 PMC3277362

[3] Acevedo BP, Poulin MJ, Collins NL, Brown LL. After the honeymoon: Neural and genetic correlates of romantic love in newlywed marriages. Front Psychol. 2020. 10.3389/fpsyg.2020.00634. 32457675 10.3389/fpsyg.2020.00634 PMC7223160

[4] Ackerman JM, Griskevicius V, Li NP. Let’s get serious: communicating commitment in romantic relationships. J Personal Soc Psychol. 2011;100(6):1079–94. 10.1037/a0022412. 10.1037/a0022412 21319910

[5] Amrhein V, Korner-Nievergelt F, Roth T. The earth is flat (*p* > 0.05): significance thresholds and the crisis of unreplicable research. PeerJ. 2017;5:e3544. 10.7717/peerj.3544. 28698825 10.7717/peerj.3544 PMC5502092

[6] Archer J. The reality and evolutionary significance of human psychological sex differences. Biol Rev Camb Philos Soc. 2019;94(4):1381–415. 10.1111/brv.12507. 30892813 10.1111/brv.12507

[7] Arnold AP. Sexual differentiation of brain and other tissues: Five questions for the next 50 years. Horm Behav. 2020. 10.1016/j.yhbeh.2020.104691. 31991182 10.1016/j.yhbeh.2020.104691 PMC7440839

[8] Bajoghli H, Keshavarzi Z, Mohammadi MR, Schmidt NB, Norton PJ, Holsboer-Trachsler E, Brand S. “I love you more than I can stand!” - Romantic love, symptoms of depression and anxiety, and sleep complaints are related among young adults. Int J Psych Clin Practice. 2014;18(3):169–74. 10.3109/13651501.2014.902072. 10.3109/13651501.2014.902072 24611539

[9] Bales K, Ardekani C, Baxter A, Karaskiewicz C, Kuske J, Lau A, Witczak L. What is a pair bond? Horm Behav. 2021;136:105062. 10.1016/j.yhbeh.2021.105062. 34601430 10.1016/j.yhbeh.2021.105062

[10] Baumard N, Huillery E, Hyafil A, Safra L. The cultural evolution of love in literary history. Nat Hum Behav. 2022. 10.1038/s41562-022-01292-z. 35256800 10.1038/s41562-022-01292-z

[11] Berner D, Amrhein V. Why and how we should join the shift from significance testing to estimation. J Evol Biol. 2022;35(6):777–87. 10.1111/jeb.14009. 35582935 10.1111/jeb.14009 PMC9322409

[12] Bode A. Romantic love evolved by co-opting mother-infant bonding. Front Psychol. 2023. 10.3389/fpsyg.2023.1176067. 37915523 10.3389/fpsyg.2023.1176067 PMC10616966

[13] Bode A, Kavanagh PS. Biological sex and romantic love among partnered, English-speaking, young adults in the first two years of being in love: a bottom-up evolutionary approach. 2022. 10.17605/OSF.IO/ACZ34

[14] Bode A, Kavanagh PS. Romantic love survey 2022. 2022. Retrieved from: 10.15139/S3/WBVMFG

[15] Bode A, Kavanagh PS. Romantic love and behavioral activation system sensitivity to a loved one. Behav Sci. 2023. 10.3390/bs13110921. 37998668 10.3390/bs13110921 PMC10669312

[16] Bode A, Kowal M. Toward consistent reporting of sample characteristics in studies investigating the biological mechanisms of romantic love. Front Psychol. 2023. 10.3389/fpsyg.2023.983419. 37213378 10.3389/fpsyg.2023.983419 PMC10192910

[17] Bode A, Kushnick G. Proximate and ultimate perspectives on romantic love. Front Psychol. 2021. 10.3389/fpsyg.2021.573123. 33912094 10.3389/fpsyg.2021.573123 PMC8074860

[18] Bode A, Kowal M, Cannas Aghedu F, Kavanagh PS. Romantic love and sexual frequency: challenging beliefs. J Sex Marital Ther. 2024. 10.1080/0092623X.2024.2394670. 39175258 10.1080/0092623X.2024.2394670

[19] Bode A, Luoto S, Kowal M, Cannas Aghedu F, Kavanagh PS. (2024b). Sexual Orientation and Romantic Love. 10.31234/osf.io/mydzu

[20] Brand S, Foell S, Bajoghli H, Keshavarzi Z, Kalak N, Gerber M, Holsboer-Trachsler E. Tell me, how bright your hypomania is, and I tell you, if you are happily in love!-Among young adults in love, bright side hypomania is related to reduced depression and anxiety, and better sleep quality. Int J Psychiatry Clin Pract. 2015;19(1):24–31. 10.3109/13651501.2014.968588. 25273225 10.3109/13651501.2014.968588

[21] Brantley A, Knox D, Zusman ME. When and why gender differences in saying I love you among college students. Coll Students J. 2002;36:614–5.

[22] Bringle RG, Winnick T, Rydell RJ. The prevalence and nature of unrequited love. SAGE Open. 2013;3(2):2158244013492160. 10.1177/2158244013492160.

[23] Buss DM. Psychological sex-differences - origins through sexual selection. Am Psychol. 1995;50(3):164–8. 10.1037/0003-066x.50.3.164. 7726470 10.1037/0003-066x.50.3.164

[24] Buss DM. The evolution of love in humans. In: Sternberg RJ, Sternberg K, editors. The new psychology of love. Second ed. Cambridge University Press; 2019.

[25] Buss DM. Evolutionary psychology is a scientific revolution. Evolutionary Behav Sci. 2020;14(4):316–23. 10.1037/ebs0000210.

[26] Buss DM, Schmitt DP. Sexual strategies theory - an evolutionary perspective on human mating. Psychol Rev. 1993;100(2):204–32. 10.1037/0033-295x.100.2.204. 8483982 10.1037/0033-295x.100.2.204

[27] Buss DM, Schmitt DP. Mate preferences and their behavioral manifestations. Annu Rev Psychol. 2019;70:77–110. 30230999 10.1146/annurev-psych-010418-103408

[28] Buss DM, Abbott M, Angleitner A, Asherian A, Biaggio A, Blancovillasenor A, Yang KS. International preferences in selecting mates - a study of 37 cultures. J Cross-Cult Psychol. 1990;21(1):5–47. 10.1177/0022022190211001.

[29] Campbell K, Hosseini C, Myers K, Calub N. Does love influence athletic performance? The perspectives of olympic athletes. Rev Euro Studies. 2016;8:1. 10.5539/res.v8n2p1. 10.5539/res.v8n2p1 PMC5703200 29187915

[30] Cannas Aghedu F, Veneziani CA, Manari T, Feybesse C, Bisiacchi PS. Assessing passionate love: Italian validation of the PLS (reduced version). Sex Relatsh Therapy. 2018;35(1):77–88. 10.1080/14681994.2018.1442570.

[31] Claessens S, Kyritsis T, Atkinson QD. Cross-national analyses require additional controls to account for the non-independence of nations. Nat Communication. 2023;14(1):5776. 10.1038/s41467-023-41486-1. 10.1038/s41467-023-41486-1 PMC10507061 37723194

[32] Climent S, Coll-Florit M. All you need is love: metaphors of love in 1946–2016 Billboard year-end number-one songs. Text Talk. 2021;41(4):469–91. 10.1515/text-2019-0209.

[33] Conroy-Beam D, Buss DM, Pham MN, Shackelford TK. How sexually dimorphic are human mate preferences? Pers Soc Psychol Bull. 2015;41(8):1082–93. 10.1177/0146167215590987. 26068718 10.1177/0146167215590987

[34] Cruces JMS, Hawrylak MF, Delegido AB. Interpersonal variability of the experience of falling in love. Int J Psychol Psychol Therapy. 2015;15(1):87–100.

[35] Cuenca-Montesino ML, Graña JL, O’Leary KD. Intensity of love in a community sample of Spanish couples in the region of Madrid. Span J Psychol. 2015;18:E79. 10.1017/sjp.2015.79. 26459102 10.1017/sjp.2015.79

[36] Darwin C. The descent of man and selection in relation to sex. John Murray; 1871.

[37] Davidian E, Surbeck M, Lukas D, Kappeler PM, Huchard E. The eco-evolutionary landscape of power relationships between males and females. Trends Ecol Evol. 2022;37(8):706–18. 10.1016/j.tree.2022.04.004. 35597702 10.1016/j.tree.2022.04.004

[38] Del Giudice M. Measuring sex differences and similarities. In: VanderLaan DP, Wong WI, editors. Gender and sexuality development. Focus on Sexuality Research. Cham: Springer; 2022. 10.1007/978-3-030-84273-4_1.

[39] Emanuele E, Politi P, Bianchi M, Minoretti P, Bertona M, Geroldi D. Raised plasma nerve growth factor levels associated with early-stage romantic love. Psychoneuroendocrinology. 2006;31(3):288–94. 10.1016/j.psyneuen.2005.09.002. 16289361 10.1016/j.psyneuen.2005.09.002

[40] Feybesse C, Hatfield E. Passionate love. In: Sternberg RJ, Sternberg K, editors. The new psychology of love. 2nd ed. Cambridge University Press; 2019.

[41] Fisher HE, Aron A, Brown LL. Romantic love: a mammalian brain system for mate choice. Philosophical Trans Royal Soc B-Biological Sci. 2006;361(1476):2173–86. 10.1098/rstb.2006.1938. 10.1098/rstb.2006.1938 PMC1764845 17118931

[42] Fletcher GJO, Simpson JA, Campbell L, Overall NC. Pair-bonding, romantic love, and evolution: the curious case of homo sapiens. Perspect Psychol Sci. 2015;10(1):20–36. 10.1177/1745691614561683. 25910380 10.1177/1745691614561683

[43] Frank RH. Passion within reason: the strategic role of the emotions. Norton; 1988.

[44] Galperin A, Haselton M. Predictors of how often and when people fall in love. Evolutionary Psychol. 2010;8(1):5–28. 10.1177/147470491000800102 PMC10481035 22947775

[45] Garcia CY. Temporal course of basic components of love along the couple relationship. Psicothema. 1997;9(1):1–15.

[46] Hackney AC, Viru A. Research methodology: endocrinologic measurements in exercise science and sports medicine. J Athl Train. 2008;43(6):631–9. 10.4085/1062-6050-43.6.631. 19030142 10.4085/1062-6050-43.6.631 PMC2582556

[47] Harrison MA, Shortall JC. Women and men in love: who really feels it and says it first? J Soc Psychol. 2011;151(6):727–36. 10.1080/00224545.2010.522626. 22208110 10.1080/00224545.2010.522626

[48] Hatfield E, Sprecher S. Measuring passionate love in intimate relationships. J Adolesc. 1986;9(4):383–410. 10.1016/S0140-1971(86)80043-4. 3805440 10.1016/s0140-1971(86)80043-4

[49] Hatfield E, Sprecher S. The Passionate Love Scale. In: Fisher TD, Davis CM, Yaber WL, Davis SL, editors. Handbook of sexuality-related measures: A compendium. 3rd ed. Taylor & Francis; 2011.

[50] Hatfield E, Walster GW. A new look at love. 1985 ed. University Press of America; 1985.

[51] Hatfield E, Bensman L, Rapson RL. A brief history of social scientists’ attempts to measure passionate love. J Social Personal Relationships. 2012;29(2):143–64. 10.1177/0265407511431055.

[52] Hendrick C, Hendrick S. A theory and method of love. J Personal Soc Psychol. 1986;50(2):392–402. 10.1037/0022-3514.50.2.392.

[53] Hendrick SS, Hendrick C. Gender differences and similarities in sex and love. Personal Relationships. 1995;2(1):55–65. 10.1111/j.1475-6811.1995.tb00077.x.

[54] Hill CA, Blakemore JEO, Drumm P. Mutual and unrequited love in adolescence and young adulthood. Personal Relationships. 1997;4(1):15–23. 10.1111/j.1475-6811.1997.tb00127.x.

[55] Janicke T, Häderer IK, Lajeunesse MJ, Anthes N. Darwinian sex roles confirmed across the animal kingdom. Sci Adv. 2016;2(2):e1500983. 10.1126/sciadv.1500983. 26933680 10.1126/sciadv.1500983 PMC4758741

[56] Jonason PK, Kavanagh PS. The dark side of love: love styles and the Dark Triad. Pers Indiv Differ. 2010;49(6):606–10. 10.1016/j.paid.2010.05.030.

[57] Kowal M, Sorokowski P, Dinić BM, Pisanski K, Gjoneska B, Frederick DA, Sternberg RJ. Validation of the short version (TLS-15) of the Triangular Love Scale (TLS-45) across 37 languages. Archiv Sex Behav. 2024. 10.1007/s10508-023-02702-7. 10.1007/s10508-023-02702-7 PMC10844340 37884798

[58] Lakens D. When and how to deviate from a preregistration. Collabra: Psychol. 2024;10(1):117094. 10.1525/collabra.117094.

[59] Langeslag SJE, van der Veen FM, Fekkes D. Blood levels of serotonin are differentially affected by romantic love in men and women. J Psychophysiol. 2012;26(2):92–8. 10.1027/0269-8803/a000071.

[60] Langeslag S, Muris P, Franken I. Measuring romantic love: Psychometric properties of the infatuation and attachment scales. J Sex Res. 2013. 10.1080/00224499.2012.714011. 23098269 10.1080/00224499.2012.714011

[61] Lewis DMG, Al-Shawaf L, Conroy-Beam D, Asao K, Buss DM. Evolutionary psychology: a how-to guide. Am Psychol. 2017;72(4):353–73. 10.1037/a0040409. 28481582 10.1037/a0040409

[62] Luoto S. An updated theoretical framework for human sexualsSelection: from ecology, genetics, and life history to extended phenotypes. Adapt Hum Behav Physiol. 2019a;5(1):48–102. 10.1007/s40750-018-0103-6.

[63] Luoto S. Response to commentaries: life gistory genetics, fluid intelligence, and extended phenotypes. Adapt Hum Behav Physiol. 2019b;5(1):112–5. 10.1007/s40750-019-0109-8.

[64] Luoto S, Varella MAC. Pandemic leadership: Sex differences and their evolutionary–developmental origins. Front Psychol. 2021. 10.3389/fpsyg.2021.633862. 33815218 10.3389/fpsyg.2021.633862 PMC8015803

[65] Luoto S, Krams I, Rantala MJ. A life history approach to the female sexual orientation spectrum: evolution, development, causal mechanisms, and health. Arch Sex Behav. 2019;48(5):1273–308. 10.1007/s10508-018-1261-0. 30229521 10.1007/s10508-018-1261-0

[66] Mahalanobis PC. A method of fractile graphical analysis. Econometrica. 1960;28(2):325–51. 10.2307/1907724.

[67] Maner JK, Ackerman JM. Ecological sex ratios and human mating. Trends Cogn Sci. 2020;24(2):98–100. 10.1016/j.tics.2019.11.008. 31892460 10.1016/j.tics.2019.11.008

[68] Marazziti D, Akiskal HS, Rossi A, Cassano GB. Alteration of the platelet serotonin transporter in romantic love. Psychol Med. 1999;29(3):741–5. 10.1017/s0033291798007946. 10405096 10.1017/s0033291798007946

[69] Marazziti D, Canale D. Hormonal changes when falling in love. Psychoneuroendocrinology. 2004;29(7):931–6. 10.1016/j.psyneuen.2003.08.006. 15177709 10.1016/j.psyneuen.2003.08.006

[70] McCarthy MM. A new view of sexual differentiation of mammalian brain. J Comp Physiol A Neuroethol Sens Neural Behav Physiol. 2020;206(3):369–78. 10.1007/s00359-019-01376-8. 31705197 10.1007/s00359-019-01376-8 PMC7196030

[71] Meltzer AL, Makhanova A, Hicks LL, French JE, McNulty JK, Bradbury TN. Quantifying the sexual afterglow: the lingering benefits of sex and their implications for pair-bonded relationships. Psychol Sci. 2017;28(5):587–98. 10.1177/0956797617691361. 28485699 10.1177/0956797617691361

[72] Meskó N, Zsidó AN, Láng A, Karádi K. Sex and relationship differences on the Short Love Attitude Scale: Insights from the Hungarian adaptation. Sexual Culture. 2021. 10.1007/s12119-021-09830-z.

[73] Meston CM, Buss DM. Why humans have sex. Arch Sex Behav. 2007;36(4):477–507. 10.1007/s10508-007-9175-2. 17610060 10.1007/s10508-007-9175-2

[74] Meston CM, Buss DM. Why women have sex. St Martin’s Griffin; 2009.

[75] Neto F. Gender differences in self-estimated types of love for self and others Interpersona. Int J Personal Relationships. 2023;17(1):130–42. 10.5964/ijpr.9297.

[76] Neto F, Pinto MdC. The role of loneliness, gender and love status in adolescents’ love styles. Int J Adolescence Youth. 2003;11(3):181–91. 10.1080/02673843.2003.9747928.

[77] Nosek BA, Ebersole CR, DeHaven AC, Mellor DT. The preregistration revolution. Proc Natl Acad Sci. 2018;115(11):2600–6. 29531091 10.1073/pnas.1708274114 PMC5856500

[78] Numan M, Young LJ. Neural mechanisms of mother-infant bonding and pair bonding: similarities, differences, and broader implications. Horm Behav. 2016;77:98–112. 10.1016/j.yhbeh.2015.05.015. 26062432 10.1016/j.yhbeh.2015.05.015 PMC4671834

[79] O’Leary KD, Acevedo BP, Aron A, Huddy L, Mashek D. Is long-term love more than a rare phenomenon? If so, what are its correlates? Soc Psychol Personality Sci. 2011;3(2):241–9. 10.1177/1948550611417015.

[80] Oliva VM. Romantic love in Disney movies. Calle 14-Revista De Investigacion En El Campo Del Arte. 2022;17(31):80–96. 10.14483/21450706.18692.

[81] Palan S, Schitter C. Prolific.ac—A subject pool for online experiments. J Behav Exp Finance. 2018;17:22–7. 10.1016/j.jbef.2017.12.004.

[82] Penny KI. Appropriate critical values when testing for a single multivariate outlier by using the mahalanobis distance. J Royal Stat Soc Ser C (Applied Statistics). 1996;45(1):73–81. 10.2307/2986224.

[83] Rogier G, Di Marzio F, Presicci C, Cavalli RG, Velotti P. Love addiction and sexual satisfaction within the attachment perspective: an empirical contribution. Psychol Sexuality. 2024. 10.1080/19419899.2024.2334412.

[84] Salk RH, Hyde JS, Abramson LY. Gender differences in depression in representative national samples: Meta-analyses of diagnoses and symptoms. Psychol Bull. 2017;143(8):783–822. 10.1037/bul0000102. 28447828 10.1037/bul0000102 PMC5532074

[85] Schacht R, Kramer KL. Are we monogamous? A review of the evolution of pair-bonding in humans and its contemporary variation cross-culturally. Front Ecol Evol. 2019. 10.3389/fevo.2019.00230.

[86] Schiefenhövel W. Romantic love. A human universal and possible honest signal. Hum Ontogenetics. 2009;3(2):39–50. 10.1002/huon.200900005.

[87] Sorokowski P, Kowal M, Sternberg RJ, Aavik T, Akello G, Alhabahba MM, Sorokowska A. Modernization, collectivism, and gender equality predict love experiences in 45 countries. Sci Rep. 2023;13(1):773. 10.1038/s41598-022-26663-4. 36641519 10.1038/s41598-022-26663-4 PMC9840424

[88] Spector IP, Carey MP, Steinberg L. The sexual desire inventory: development, factor structure, and evidence of reliability. J Sex Marital Ther. 1996;22(3):175–90. 10.1080/00926239608414655. 8880651 10.1080/00926239608414655

[89] Stephen I, Luoto S. Physical cues of partner quality. In: Mogilski J, Shackelford T, editors. The Oxford handbook of evolutionary psychology and romantic relationships. Oxford University Press; 2022. 10.1093/oxfordhb/9780197524718.013.2.

[90] Sternberg RJ. A triangular theory of love. Psychol Rev. 1986;93(2):119–35. 10.1037/0033-295x.93.2.119.

[91] Sternberg RJ. Construct validation of a triangular love scale. Eur J Social Psychol. 1997;27(3):313–35.

[92] Stewart-Williams S, Thomas AG. The ape that thought it was a peacock: does evolutionary psychology exaggerate human sex differences? Psychol Inq. 2013;24(3):137–68. 10.1080/1047840X.2013.804899.

[93] Tehrani HD, Yamini S. Gender differences concerning love: a meta-analysis. TPM-Testing Psychometrics Methodol Appl Psychol. 2020;27(4):603–26.

[94] Tooby J. Evolutionary psychology as the crystalizing core of a unified modern social science. Evol Behav Sci. 2020;14:390–403.

[95] Tooby J, Cosmides L, Barrett HC. The second law of thermodynamics is the first law of psychology: evolutionary developmental psychology and the theory of tandem, coordinated inheritances: comment on Lickliter and Honeycutt (2003). Psychol Bull. 2003;129(6):858–65. 10.1037/0033-2909.129.6.858. 14599284 10.1037/0033-2909.129.6.858

[96] Trivers RL. Parental investment and sexual selection. In: Campbell B, editor. Sexual selection and the descent of man. UK: Aldine; 1972. p. 136–79.

[97] United Nations Department of Eocnonic and Soocial Affairs Population Division. World Population Prospects 2022, Online Edition. 2022. https://www.un.org/development/desa/pd/sites/www.un.org.development.desa.pd/files/wpp2022_summary_of_results.pdf

[98] United Nations Development Program. (2022). Human Development Report 2021-22: Uncertain times, unsettled lives: Shaping our future in a transforming world. Retrieved from https://hdr.undp.org/content/human-development-report-2021-22

[99] Van de Vliert E, Van Lange PAM. Latitudinal psychology: an ecological perspective on creativity, aggression, happiness, and beyond. Perspect Psychol Sci. 2019;14(5):860–84. 10.1177/1745691619858067. 31433723 10.1177/1745691619858067

[100] Walter KV, Conroy-Beam D, Buss DM, Asao K, Sorokowska A, Sorokowski P, Zupančič M. Sex differences in mate preferences across 45 countries: A large-scale replication. Psychol Sci. 2020. 10.1177/0956797620904154. 32196435 10.1177/0956797620904154

[101] Walter K, Conroy-Beam D, Buss D, Asao K, Sorokowska A, Sorokowski P, Batres C. (2021). Sex differences in human mate preferences vary across sex ratios. Proceedings of the Royal Society of London. Series B, Containing papers of a Biological character. Royal Society (Great Britain), 288, 20211115. 10.1098/rspb.2021.1115 PMC8292757 34284630

[102] Watkins C, Bovet J, Fernandez AM, Leongómez JD, Zelazniewicz A, Correa Varella MA, Wagstaff DL. Men say I love you before women do: Robust across several countries. J Soc Personal Relationships. 2022. 10.1177/02654075221075264.

[103] Zhu N, Chang L. Evolved but Not Fixed: A life history account of gender toles and gender inequality. Front Psychol. 2019. 10.3389/fpsyg.2019.01709. 31396136 10.3389/fpsyg.2019.01709 PMC6664064

