# Supplementary material for: Sex differences in romantic love: an evolutionary perspective
Source: Biol Sex Differ. 2025 Feb 24;16:16. doi: 10.1186/s13293-025-00698-4 (PMC11849325; doi:10.1186/s13293-025-00698-4)
Supplement: Supplementary file 1 — Supplementary material 1 [file 13293_2025_698_MOESM1_ESM.docx]

# Supplementary Material

**Supplementary Table 1.**

*Sample Characteristics*

| Variables | Range | *M* | *SD* | *Md* | *n* | % |
| --- | --- | --- | --- | --- | --- | --- |
| Sample |  |  |  |  | 808 | 100.00 |
| Female |  |  |  |  | 386 | 47.77 |
| Heterosexual |  |  |  |  | 589 | 72.90 |
| Age | 18.00, 25.00 | 22.16 | 1.86 | 22.00 |  |  |
| PLS-30 (mean item; 1-9) | 4.37, 9.00 | 6.99 | 1.04 | 7.10 |  |  |
| TLS- Commitment (mean item; 1-9) | 2.00, 9.00 | 7.32 | 1.37 | 7.60 |  |  |
| Percent of day thinking about loved one | 2.00, 100.00 | 49.04 | 22.58 | 49.00 |  |  |
| Relationship status |  |  |  |  |  |  |
| Dating, not co-habiting |  |  |  |  | 357 | 44.18 |
| Committed, not co-habiting |  |  |  |  | 371 | 45.92 |
| Committed, co-habiting |  |  |  |  | 74 | 9.16 |
| Married or de facto |  |  |  |  | 6 | 0.74 |
| Satisfaction with relationship (1-5) | 1.00, 5.00 | 4.18 | 0.78 | 4.00 |  |  |
| Time in love (months) | 0.00, 23.00 | 8.12 | 5.97 | 7.00 |  |  |
| Relationship duration (months) | 0.00, 46.00 | 9.55 | 7.07 | 8.00 |  |  |
| Having sex |  |  |  |  | 719 | 88.99 |
| Times sex per week | 0.00, 20.00 | 3.21 | 2.87 | 3.00 |  |  |
| >13 years of education |  |  |  |  | 690 | 85.40 |
| Current student |  |  |  |  | 565 | 69.93 |
| Employment |  |  |  |  |  |  |
| No |  |  |  |  | 353 | 43.69 |
| Less than full-time |  |  |  |  | 275 | 34.03 |
| Full-time |  |  |  |  | 180 | 22.28 |
| AQOL-4D (12-48) | 12.00, 35.00 | 17.55 | 3.47 | 17.00 |  |  |
| Note: PLS-30 = Passionate Love Scale; TLS-Commitment = the 5 items from the Triangular Love Scale identified by (Kowal et al., 2024) as forming part the TLS-15 (the survey used a 9-point measurements scale instead of a five-point scale for all items); AQOL-4D = The Assessment of Quality of Life – 4D; The range, mean, SD, and median for the PLS-30 and TLS Commitment were divided by the number of items to maximize comparability with other studies. A small number of cases are missing from the continuous variables because of missing data. | | | | | | |

**Supplementary Table 2.**

*Countries in Which Participants Resided*

| **Country** | ***n*** | **%** |
| --- | --- | --- |
| South Africa | 101 | 12.50 |
| Poland | 71 | 8.79 |
| United Kingdom of Great Britain and Northern Ireland | 59 | 7.30 |
| Portugal | 57 | 7.05 |
| Mexico | 56 | 6.93 |
| Greece | 50 | 6.19 |
| Germany | 49 | 6.06 |
| Spain | 49 | 6.06 |
| Italy | 47 | 5.82 |
| United States of America | 39 | 4.83 |
| Hungary | 38 | 4.70 |
| Netherlands | 31 | 3.84 |
| Canada | 22 | 2.72 |
| Chile | 22 | 2.72 |
| France | 16 | 1.98 |
| Slovenia | 14 | 1.73 |
| Estonia | 11 | 1.36 |
| Australia | 10 | 1.24 |
| Czech Republic | 10 | 1.24 |
| Latvia | 9 | 1.11 |
| Austria | 8 | 0.99 |
| Rest of sample | 39 | 4.83 |
| Note. Rest of sample = Finland, Ireland, Israel, New Zealand, Belgium, Sweden, Switzerland, Japan, South Korea, Denmark, Luxembourg, Norway | | |

**Supplementary Table 3**

*Hierarchical Regression Model of Love Progression with an Additional Love-related Variable as a Control*

|  |  |  |  |  |  |  |  |  | 95% *CI* | |
| --- | --- | --- | --- | --- | --- | --- | --- | --- | --- | --- |
|  | *R^2^* | Adjusted *R^2^* | Δ Adjusted *R^2^* | *b* | *SE* | β | *t* | *p* | Lower | Upper |
| Step 1 | .034*** | .029*** |  |  |  |  |  |  |  |  |
| Age |  |  |  | 0.177 | 0.082 | .075 | 2.16 | .031 | 0.02 | 0.34 |
| Sex ratio |  |  |  | -0.009 | 0.051 | -.007 | -0.18 | .854 | -0.11 | 0.09 |
| Gender inequality |  |  |  | 1.052 | 1.330 | .029 | 0.79 | .429 | -1.56 | 3.66 |
| Number of times in love |  |  |  | -0.524 | 0.105 | -.175 | -4.99 | <.001 | -0.73 | -0.32 |
| Step 2 | .041*** | .035*** | .006* |  |  |  |  |  |  |  |
| Age |  |  |  | 0.164 | 0.082 | .069 | 2.00 | .046 | 0.00 | 0.32 |
| Sex ratio |  |  |  | -0.004 | 0.050 | -.002 | -0.07 | .945 | -0.10 | 0.10 |
| Gender inequality |  |  |  | 1.028 | 1.326 | .028 | 0.78 | .438 | -1.57 | 3.63 |
| Number of times in love |  |  |  | -0.494 | 0.105 | -.165 | -4.69 | <.001 | -0.70 | -0.29 |
| Biological sex (male) |  |  |  | -0.747 | 0.306 | -.085 | -2.44 | .015 | -1.35 | -0.15 |
| Note. *n* = 808; ^*^*p* < .05; ^***^*p* < .001 | | | | | | | | | | |

**Supplementary Table 4**

*Hierarchical Regression Model of Intensity of Romantic Love with Additional Romantic Love-related Variables as Controls*

|  |  |  |  |  |  |  |  |  | 95% *CI* | |
| --- | --- | --- | --- | --- | --- | --- | --- | --- | --- | --- |
|  | *R^2^* | Adjusted *R^2^* | Δ Adjusted *R^2^* | *b* | *SE* | β | *t* | *p* | Lower | Upper |
| Step 1 | .459*** | .453*** |  |  |  |  |  |  |  |  |
| Age |  |  |  | -0.412 | 0.442 | -.025 | -0.93 | .352 | -1.28 | 0.46 |
| Sex ratio |  |  |  | 0.248 | 0.270 | .025 | 0.92 | .360 | -0.28 | 0.78 |
| Gender inequality |  |  |  | 5.713 | 7.236 | .022 | 0.79 | .430 | -8.49 | 19.92 |
| Number of times in love |  |  |  | -0.757 | 0.581 | -.036 | -1.30 | .193 | -1.90 | 0.38 |
| Love progression |  |  |  | -0.410 | 0.191 | -.058 | -2.15 | .032 | -0.78 | -0.03 |
| Months in love |  |  |  | -0.008 | 0.142 | -.002 | -0.06 | .956 | -0.29 | 0.27 |
| Obsessive thinking |  |  |  | 0.412 | 0.039 | .298 | 10.60 | <.001 | 0.34 | 0.49 |
| Commitment |  |  |  | 2.301 | 0.132 | .503 | 17.43 | <.001 | 2.04 | 2.56 |
| Days since sex |  |  |  | 0.508 | 0.285 | .047 | 1.78 | .075 | -0.05 | 1.07 |
| Step 2 | .460*** | .453*** | .000 |  |  |  |  |  |  |  |
| Age |  |  |  | -0.429 | 0.442 | -.026 | -0.97 | .333 | -1.30 | 0.44 |
| Sex ratio |  |  |  | 0.256 | 0.271 | .025 | 0.95 | .345 | -0.28 | 0.78 |
| Gender inequality |  |  |  | 5.900 | 7.242 | .022 | 0.81 | .415 | -8.32 | 20.12 |
| Number of times in love |  |  |  | -0.726 | 0.583 | -.034 | -1.25 | .213 | -1.87 | 0.42 |
| Love progression |  |  |  | -0.425 | 0.192 | -.060 | -2.21 | .027 | -0.80 | -0.05 |
| Months in love |  |  |  | -0.013 | 0.142 | -.002 | -0.09 | .927 | -0.29 | 0.27 |
| Obsessive thinking |  |  |  | 0.406 | 0.040 | .294 | 10.24 | <.001 | 0.33 | 0.48 |
| Commitment |  |  |  | 2.301 | 0.132 | .503 | 17.43 | <.001 | 2.04 | 2.56 |
| Days since sex |  |  |  | 0.503 | 0.285 | .046 | 1.76 | .078 | -0.06 | 1.06 |
| Biological sex (male) |  |  |  | -1.272 | 1.686 | -.020 | -0.75 | .451 | -4.58 | 2.04 |

Note. *n* = 808; ^**^*p* < .01; ^***^*p* < .001

**Supplementary Table 5**

*Hierarchical Regression Model of Obsessive Thinking without Additional Romantic Love-related Variables as Controls*

|  |  |  |  |  | |  | |  | | 95% *CI* | | |  |
| --- | --- | --- | --- | --- | --- | --- | --- | --- | --- | --- | --- | --- | --- |
|  | *R^2^* | Adjusted *R^2^* | Δ Adjusted *R^2^* | *b* | *SE* | | β | | *t* | | Lower | Upper | |
| Step 1 | .248 | .240 |  |  |  | |  | |  | |  |  | |
| Age |  |  |  | 0.357 | 0.376 | | .029 | | 0.95 | | -0.38 | 1.10 | |
| Sex ratio |  |  |  | -0.257 | 0.230 | | -.035 | | -1.12 | | -0.71 | 0.20 | |
| Gender inequality |  |  |  | 24.017 | 6.110 | | .127 | | 3.93 | | 12.02 | 36.01 | |
| Number of times in love |  |  |  | -0.716 | 0.495 | | -.047 | | -1.45 | | -1.69 | 0.26 | |
| Love progression |  |  |  | 0.020 | 0.163 | | .004 | | 0.12 | | -0.30 | 0.34 | |
| Months in love |  |  |  | -0.103 | 0.121 | | -.027 | | -0.85 | | -0.34 | 0.13 | |
| Intensity of romantic love |  |  |  | 0.299 | 0.028 | | .414 | | 10.60 | | 0.24 | 0.35 | |
| Commitment |  |  |  | 0.230 | 0.132 | | .070 | | 1.74 | | -0.03 | 0.49 | |
| Days since sex |  |  |  | -0.307 | 0.243 | | -.039 | | -1.26 | | -0.78 | 0.17 | |
| Step 2 | .271 | .261 | .021 |  |  | |  | |  | |  |  | |
| Age |  |  |  | 0.251 | 0.371 | | .021 | | 0.68 | | -.048 | 0.98 | |
| Sex ratio |  |  |  | -0.203 | 0.227 | | -.028 | | -0.90 | | -.065 | 0.24 | |
| Gender inequality |  |  |  | 24.337 | 6.022 | | .128 | | 4.04 | | 12.52 | 36.16 | |
| Number of times in love |  |  |  | -0.530 | 0.489 | | -.034 | | -1.08 | | -1.49 | 0.43 | |
| Love progression |  |  |  | -0.065 | 0.162 | | -.013 | | -0.40 | | -0.38 | 0.25 | |
| Months in love |  |  |  | -0.127 | 0.119 | | -.034 | | -1.07 | | -0.36 | 0.11 | |
| Intensity of romantic love |  |  |  | 0.286 | 0.028 | | .396 | | 10.24 | | 0.23 | 0.34 | |
| Commitment |  |  |  | 0.232 | 0.130 | | .070 | | 1.79 | | -0.02 | 0.49 | |
| Days since sex |  |  |  | -0.323 | 0.240 | | -.041 | | -1.35 | | -0.79 | 0.15 | |
| Biological sex (male) |  |  |  | -6.924 | 1.395 | | -.153 | | -4.97 | | -9.66 | -4.19 | |

Note. *n* = 808

**Supplementary Table 6**

*Hierarchical Regression Model of Commitment without Additional Romantic Love-related Variables as Controls*

|  |  |  |  |  |  |  |  | 95% CI | |  |
| --- | --- | --- | --- | --- | --- | --- | --- | --- | --- | --- |
|  | *R^2^* | Adjusted *R^2^* | Δ Adjusted *R^2^* | *b* | *SE* | β | *t* | Lower | Upper | |
| Step 1 | 0.411 | 0.405 |  |  |  |  |  |  |  | |
| Age |  |  |  | -0.043 | 0.101 | -.012 | -0.43 | -0.24 | 0.16 | |
| Sex ratio |  |  |  | -0.028 | 0.062 | -.013 | -0.46 | -0.15 | 0.09 | |
| Gender inequality |  |  |  | 1.063 | 1.652 | .019 | 0.64 | -2.18 | 4.31 | |
| Number of times in love |  |  |  | -0.255 | 0.132 | -.055 | -1.92 | -0.51 | 0.01 | |
| Love progression |  |  |  | -0.045 | 0.044 | -.029 | -1.04 | -0.13 | 0.04 | |
| Months in love |  |  |  | 0.171 | 0.032 | .149 | 5.36 | 0.11 | 0.23 | |
| Intensity of romantic love |  |  |  | 0.120 | 0.007 | .548 | 17.43 | 0.11 | 0.13 | |
| Obsessive thinking |  |  |  | 0.017 | 0.009 | .055 | 1.74 | 0.00 | 0.04 | |
| Days since sex |  |  |  | -0.221 | 0.065 | -.093 | -3.42 | -0.35 | -0.09 | |
| Step 2 | 0.412 | 0.404 | 0.000 |  |  |  |  |  |  | |
| Age |  |  |  | -0.041 | 0.101 | -.011 | -0.41 | -0.24 | 0.16 | |
| Sex ratio |  |  |  | -0.029 | 0.062 | -.013 | -0.47 | -0.15 | 0.09 | |
| Gender inequality |  |  |  | 1.039 | 1.654 | .018 | 0.63 | -2.21 | 4.29 | |
| Number of times in love |  |  |  | -0.258 | 0.133 | -.055 | -1.94 | -0.52 | 0.00 | |
| Love progression |  |  |  | -0.043 | 0.044 | -.028 | -0.99 | -0.13 | 0.04 | |
| Months in love |  |  |  | 0.171 | 0.032 | .150 | 5.38 | 0.11 | 0.23 | |
| Intensity of romantic love |  |  |  | 0.120 | 0.007 | .548 | 17.43 | 0.11 | 0.13 | |
| Obsessive thinking |  |  |  | 0.017 | 0.010 | .057 | 1.79 | 0.00 | 0.04 | |
| Days since sex |  |  |  | -0.221 | 0.065 | -.093 | -3.41 | -0.35 | -0.09 | |
| Biological sex (male) |  |  |  | 0.157 | 0.385 | .012 | 0.41 | -0.60 | 0.91 | |

Note. *n* = 808
